# Genomic instability at the locus of sterol C24-methyltransferase promotes amphotericin B resistance in *Leishmania* parasites

**DOI:** 10.1371/journal.pntd.0007052

**Published:** 2019-02-04

**Authors:** Andrew W. Pountain, Stefan K. Weidt, Clément Regnault, Paul A. Bates, Anne M. Donachie, Nicholas J. Dickens, Michael P. Barrett

**Affiliations:** 1 Wellcome Centre for Molecular Parasitology, Institute of Infection, Immunity & Inflammation, University of Glasgow, Glasgow, United Kingdom; 2 Department of Microbiology and Molecular Genetics, University of Texas Health Science Center at Houston, Houston, Texas, United States of America; 3 Glasgow Polyomics, College of Medical, Veterinary & Life Sciences, University of Glasgow, Garscube Estate, Bearsden, Glasgow, United Kingdom; 4 Division of Biomedical and Life Sciences, Faculty of Health and Medicine, Lancaster University, United Kingdom; 5 Marine Biomedical & Biotechnology Research Program, Florida Atlantic University Harbor Branch Oceanographic Institute, Fort Pierce, Florida, United States of America; Northeastern University, UNITED STATES

## Abstract

Amphotericin B is an increasingly important tool in efforts to reduce the global disease burden posed by *Leishmania* parasites. With few other chemotherapeutic options available for the treatment of leishmaniasis, the potential for emergent resistance to this drug is a considerable threat. Here we characterised four novel amphotericin B-resistant *Leishmania mexicana* lines. All lines exhibited altered sterol biosynthesis, and hypersensitivity to pentamidine. Whole genome sequencing demonstrated resistance-associated mutation of the sterol biosynthesis gene sterol C5-desaturase in one line. However, in three out of four lines, RNA-seq revealed loss of expression of sterol C24-methyltransferase (SMT) responsible for drug resistance and altered sterol biosynthesis. Additional loss of the miltefosine transporter was associated with one of those lines. SMT is encoded by two tandem gene copies, which we found to have very different expression levels. In all cases, reduced overall expression was associated with loss of the 3’ untranslated region of the dominant gene copy, resulting from structural variations at this locus. Local regions of sequence homology, between the gene copies themselves, and also due to the presence of SIDER1 retrotransposon elements that promote multi-gene amplification, correlate to these structural variations. Moreover, in at least one case loss of SMT expression was not associated with loss of virulence in primary macrophages or *in vivo*. Whilst such repeat sequence-mediated instability is known in *Leishmania* genomes, its presence associated with resistance to a major antileishmanial drug, with no evidence of associated fitness costs, is a significant concern.

## Author summary

*Leishmania* parasites are a significant threat, causing some 0.9–1.6 million new infections and 20,000 deaths annually. Amphotericin B is gaining importance in control of leishmaniasis, as other treatment options are increasingly compromised by emergent drug resistance. We sought to identify mechanisms by which resistance to amphotericin B could emerge through experimental selection and characterisation of four novel resistant *L*. *mexicana* lines. Whilst in all cases, sterol metabolism was dramatically affected, in three lines specific sterol changes resulted from decreased expression of the sterol biosynthesis enzyme sterol C24-methyltransferase (SMT). We found that this resulted from structural variation events at the genome level, resulting in specific loss of the more highly expressed SMT gene copy. These events appear to arise due to repetitive sequence elements in the genomic region, suggesting that SMT could be particularly prone to loss of expression in this manner. Moreover, in at least one line, we found that disruption of SMT function was not associated with any loss of fitness. These findings are significant in demonstrating that an apparently common mechanism of resistance-associated mutation does not compromise parasite virulence, thus representing a possible route to emergent resistance in the clinical setting.

## Introduction

Protozoan *Leishmania* parasites represent a significant burden to global health [1]. Manifestations of leishmaniasis range from disabling and disfiguring cutaneous and mucocutaneous forms to lethal visceral leishmaniasis. Control is dependent on a handful of chemotherapeutic options [2], with pentavalent antimony-based compounds the treatment of choice for over seventy years. In recent decades, the toxicity of these compounds and the emergence of resistance has increased pressure to adopt alternatives [3]. Miltefosine was introduced initially to the Indian subcontinent in 2002, but treatment efficacy rates appear to have declined [4,5] and resistant strains have been isolated [6,7].

In this context, amphotericin B (AmB) is gaining importance for the treatment of leishmaniasis [8]. The introduction of liposomal forms [9–11] has substantially improved safety and efficacy profiles. Single injections of a liposomal formulation, AmBisome, reduce costs and greatly simplify treatment protocols [10]. However, this minimalist treatment strategy carries the risk of selecting AmB resistance. Several cases of treatment failure in humans have been reported [12–15] and in at least one case AmB resistant parasites were isolated [15]. Understanding the routes by which resistance may emerge is thus an important priority.

AmB exhibits specific cidal activity against *Leishmania*, as well as fungi, through interaction with ergosterol and close structural relatives, the major membrane sterol components in these organisms. Binding to cholesterol, the primary mammalian membrane sterol, is less avid due to its lacking conjugated 5(6)-7(8) double bonds in the ring structure (cholesterol has only a 5(6) double bond), and also methylation at the C24 position found in ergosterol [16,17]. Selection for AmB resistance in *Leishmania* in the cultured promastigote form has revealed loss of ergosterol-like sterols and accumulation of intermediates lacking one or both of these features in resistant lines [18–21].

Previously, we demonstrated a role for mutation in the sterol biosynthesis enzyme, lanosterol 14α-demethylase (CYP51), in AmB resistance in an *L*. *mexicana* line [21]. Sterol C24-methyltransferase (SMT), which introduces the C24-methyl group within the ergosterol side chain, has been implicated since loss of C24-methylation has been noted in multiple AmB-resistant *Leishmania* lines [15,18]. Furthermore, AmB-resistant *L*. *donovani* displaying altered SMT transcript expression has been documented [22] where one of two distinct SMT transcripts detected in wild-type parasites is lost in resistant lines. Similar changes were observed in an *L*. *donovani* line isolated from an AmB non-responsive patient, accompanied by loss of C24-methylation and accumulation of cholesta-5,7,24-trienol [15].

Here we show, in three independently selected AmB-resistant *L*. *mexicana* lines, that such changes can arise through structural variations at the genomic locus of SMT. We provide evidence that structural instability may be promoted by tandem homologous sequences, and that such disruptions do not necessarily entail fitness costs, despite having a large influence on sterol composition. We also present, for the first time, mutation in sterol C5-desaturase in a fourth line, as well as loss of function in the miltefosine transporter contributing to resistance in a sterol-independent fashion, as has been described previously [20]. However, the apparent propensity of the SMT locus for structural variation is of particular concern, and provides an important example of how copy number instability in *Leishmania* genomes contributes to resistance to an antileishmanial drug of growing importance.

## Methods

### Selection of AmB-resistant promastigotes

*Leishmania mexicana* strain M379 promastigotes were cultured in HOMEM (GE Healthcare) supplemented with 10% v/v fetal bovine serum (FBS, Gibco). Selection of drug resistance was achieved by continuous culture in stepwise increasing AmB (Sigma), starting at 50 nM and rising to 400 nM in two lines (AmBRA and AmBRB) and 200 nM in two (AmBRC and AmBRD). Each time parasites were subcultured they were diluted to 1 x 10^6^ cells/ml in 10 ml, meaning that 10^7^ parasites were exposed to drug. During selection, the medium was supplemented with 100 units/ml penicillin and 0.1 mg/ml streptomycin. Individual clones were obtained by limiting dilution.

### Drug sensitivity assays

Drug sensitivity in promastigotes was determined using the Alamar Blue assay [23], with the following modifications. Mid-log phase parasites were exposed to serial dilutions of the active compound at a starting density of 10^6^ parasites/ml at 25 ^o^C for 72 hours, before resazurin solution (Sigma) was added to a final concentration of 4.5 μM. After incubating for a further 48 hours at 25 ^o^C, fluorescence was measured using a FLUOstar OPTIMA microplate reader (BMG Labtech) with excitation and emission wavelengths of 530 nM and 590 nM, respectively. The concentration at which 50% growth inhibition was achieved (IC_50_) was calculated using the R package drc version 2.5 [24], with a four parameter dose-response model. Significance was tested using the two-tailed student’s *t* test. Error bars on IC_50_ plots depict standard error. All compounds were purchased from Sigma, except for pentamidine isethionate, which was from May & Baker. Sample number (n) values relate to independent biological replicates, with each biological replicate an average of two technical replicates.

### Sterol analysis

Mid-log phase promastigotes were washed once with PBS and resuspended in 25% m/v KOH dissolved in 3:2 v/v ethanol:water, followed by heating at 85 ^o^C for 1 hour. After cooling, samples were partitioned by vortexing for 30 s with an equal volume of *n*-heptane (Sigma). After allowing layers to separate for 20 min, the upper organic layer was retained as the sterol extract. For gas chromatography-mass spectrometry (GC-MS, performed at Glasgow Polyomics), sterol was extracted from 3 x 10^8^ parasites in 500 μL *n*-heptane. Trimethylsilane derivatisation was performed by adding 50 μl MSTFA + 1% TMCS (Thermo Scientific) to dried samples, vortexing and incubation at 80 ^o^C for 15 min. A retention index was added. GC was performed using 1.0 ml/min Helium carrier gas in a TraceGOLD TG-5SILMS column (30 m length, 0.25 mm inner diameter, 0.25 μm film thickness, Thermo Scientific) installed in a Trace Ultra gas chromatograph (Thermo Scientific). Derivatised sample (1 μl) was injected into a split/splitless injector using a surged splitless injection (splitless time 30 s, surge pressure 167 kPa). An initial oven temperature of 70 ^o^C was increased to 250 ^o^C at a ramp rate of 50 ^o^C/min; this was reduced to 10 ^o^C/min, with a final temperature of 330 ^o^C then held for 3.5 min. Eluting peaks were transferred at an auxiliary transfer temperature of 250 ^o^C to an ITQ900-GC mass spectrometer (Thermo Scientific), with an emission current of 50 μA. The ion source was held at 230 ^o^C, the full scan mass range was 50–700 *m/z* with an automatic gain control of 50% and maximum ion time of 50 ms. Blank samples were prepared by heating alcoholic KOH and partitioning with *n*-heptane. Quality control samples were prepared by pooling equal volumes of all samples. Analysis was performed using TraceFinder v3.3 (Thermo Scientific), with identification either by match to a panel of standards or by comparison to the National Institute of Standards and Technology (NIST) library, also using TraceFinder. After calculating initial percentage abundance for different identified peaks, those with a relative abundance of < 0.5% were omitted and percentages recalculated to give the final values shown. For UV spectroscopy, sterol extracts from 8 x 10^7^ cells were measured using a Shimadzu UV-2550 UV-Vis spectrophotometer and UVProbe v2.34 software, using *n*-heptane as a blank. An ergosterol standard spectrum was obtained using 0.05 mg/ml ergosterol (Sigma) in *n*-heptane. Biological replicates used relate to sterol extracts prepared independently from separate biological samples.

### High throughput sequencing analysis

Genomic DNA and total RNA were extracted using Nucleospin Tissue and Nucleospin RNA kits, respectively (both Macherey-Nagel). For whole genome sequencing (WGS), samples were prepared using the TruSeq Nano DNA library preparation kit (Illumina). For RNA-seq the TruSeq stranded mRNA library preparation kit (Illumina) was used according to manufacturer’s protocol except that initial fragmentation was performed using a Bioruptor Pico (Diagenode). Sequencing was performed at Glasgow Polyomics, obtaining 2 x 75 bp paired-end reads using a NextSeq 500 sequencer (Illumina), although for the sequencing data for AmB-resistant amastigotes, reads were obtained using both NextSeq 500 and HiSeq 4000 sequencers (Illumina). For data analysis, adaptor removal and quality trimming were performed using Trim Galore! (Babraham Bioinformatics) and reads were aligned to the *L*. *mexicana* MHOM/GT/2001/U1103 reference genome release 9.0, using BWA v2.2.1 [25] (applicable for RNA-seq due to the lack of introns in *Leishmania*), with further processing using Samtools v1.2 [26] and Picard Tools v1.138 (http://broadinstitute.github.io/picard/). Per-gene mapped fragment counts were calculated using HTSeq-count v0.6.1 [27]. For WGS, chromosomal ploidy was calculated as a ratio between the median length-normalised mapped fragment count for an individual chromosome, compared to the median across all chromosomes. Variant detection was performed using Freebayes v1.1.0 [28]. Mapped read counts were determined for windows of defined length (100 bp or 500 bp as stated) using Bedtools v2.19.1 (http://bedtools.readthedocs.io/), and normalised by a ratio of total mapped reads across the parental chromosome to that of wild-type. For RNA-seq, normalisation and differential expression analysis was performed using DESeq2 v1.14.0 [29]. All raw reads data generated in high throughput sequencing experiments have been submitted to the European Nucleotide Archive (https://www.ebi.ac.uk/ena/) with project references PRJEB23762 (WGS) and PRJEB23867 (RNA-seq).

### Quantitative PCR

Primers were designed using Primer Express v3 (Applied Biosystems) (S6 Table). For qRT-PCR, total RNA was extracted using the Nucleospin RNA kit (Macherey Nagel) according to manufacturer’s instructions except a longer DNase treatment step of 30 min was used. Reverse transcription was carried out using Superscript III reverse transcriptase (Invitrogen) for 60 min, followed by RNA degradation with RNase H (Invitrogen). Quantitative PCR was performed using Power SYBR Green PCR master mix (Applied Biosystems) according to the manufacturer’s protocol in MicroAmp Optical 96-well reaction plates (Applied Biosystems), with a primer concentration of 300 nM. Measurement was in a 7500 real-time PCR system Applied Biosystems) according to the following protocol: 2 min at 50 ^o^C, 10 min at 95°C, then 40 cycles of 95 ^o^C, 15 s and 60 ^o^C, 60 s. ROX dye was used as a passive reference. All cycle threshold (Ct) values derived were the average of three technical replicates. As well as water controls, reverse transcriptase (RT)-free controls were used in all cases, with a δCt between +RT and–RT of six deemed sufficient (in two samples of infected macrophages, this δCt was only five, but this was likely due to low parasite material due to poor infection rates, and was thus tolerated). RNA from uninfected macrophages did not lead to specific amplification Ct > 34) for any primers used for intracellular amastigotes. Normalisation was to the cytosolic glyceradehyde 3-phosphate dehydrogenase gene (GAPDH, *LmxM*.*36*.*2350*). This has been used for this purpose previously [30,31], and is a single-copy gene which has unaltered expression both in RNA-seq data presented here, and in a previously published dataset comparing *L*. *mexicana* promastigotes and amastigotes [32]. Moreover, no ploidy changes were observed in its parent chromosome (LmxM.20) in any resistant line. For qPCR on genomic DNA, reactions were performed as above, except that normalisation was achieved on the basis of sample loading alone (5 ng DNA per reaction). Where tests of significance were performed, these were performed on δCt values using a two-tailed student’s *t* test. Error bars in plots depict standard deviation in fold-change. Biological replicates were obtained using total RNA or genomic DNA extracts from independent biological samples.

### Generation of episomal overexpression lines

Genes of interest were amplified from genomic DNA (primers in S7 Table) using Phusion polymerase (New England Biolabs) according to manufacturer’s protocol with 3% DMSO added. The thermocycling protocol was as follows: 98 ^o^C for 30 s; 30 cycles of denaturation at 98 ^o^C, 10 s, annealing for 30 s (63 ^o^C for *LmxM*.*23*.*1300*, 67.3 ^o^C for *LmxM*.*13*.*1530*, 72 ^o^C for SMT), and elongation at 72 ^o^C (30 s for *LmxM*.*23*.*1300* and SMT, 90 s for *LmxM*.*13*.*1530*); 10 min final elongation at 72 ^o^C. Amplicons were inserted into the pNUS vector pGL1132 after linearisation and excision of GFP (using restriction enzymes BglII and XhoI in NEBuffer 3.1, all from New England Biolabs) using the NEBuilder ligation-free cloning system (New England Biolabs) according to the manufacturer’s protocol. Vector DNA isolated from clones was purified by ethanol precipitation. *Leishmania* lines were prepared for transfection by resuspending in ice-cold transfection buffer (90 mM Na_2_HPO_4_, 90 mM NaH_2_PO_4_, 5 mM KCl, 50 mM HEPES, 0.15 mM CaCl_2_, pH 7.3). After addition of plasmid, transfection by electroporation was performed using an Amaxa Nucleofector II (Lonza) on program U-033. Cells were incubated in culture medium at 25 ^o^C overnight, before addition of 25 μg/ml G418 selection marker (Sigma). Transfected lines were maintained continuously in the presence of G418, with selection for at least a week prior to use (during drug assays with transfected lines, 12.5 μg/ml G418 was used). Correct insert sequences were verified using sequencing primers in S8 Table.

### Sequencing of genomic regions

PCR amplicons were prepared for sequencing using the Nucleospin gel and PCR cleanup kit (Macherey-Nagel) and Sanger sequencing of samples was performed by Eurofins Genomics. PCR amplification of the intergenic region used forward and reverse primers at the 3’- and 5’-ends of the SMT coding sequence (sequences in S7 Table), using Q5 polymerase (New England Biolabs) according to manufacturer’s protocol. The thermocycling program was as described for Phusion polymerase above, with a 60 ^o^C annealing temperature and 2 min extension time. This amplicon was cloned using the pGEM-T Easy vector system (Promega) according to manufacturer’s protocol, transforming into competent *E*. *coli* DH5α cells. Sequencing of four cloned plasmids using the primers described (S8 Table) allowed construction of a consensus sequence for the intergenic region. Sequencing of individual SMT coding sequences involved PCR amplification using the forward primer for SMT cloning and the reverse qPCR primers for *LmxM*.*36*.*2380* and *LmxM*.*36*.*2390*, using Q5 polymerase as above (annealing temperature 58 ^o^C, extension time 60 s). For amplification between the genes *LmxM*.*36*.*2540* and SMT, the same primers were used as for qPCR (forward for *LmxM*.*36*.*2540*, reverse for SMT), with Q5 polymerase as described above, with an extension time of 2.5 min and an annealing temperature of 70 ^o^C.

### Macrophage and mouse infection experiments

All mice used were purchased from Harlan and kept at the Central Research Facilities, University of Glasgow, Glasgow U.K. Primary bone marrow-derived macrophages were isolated and differentiated according to the method of Weischenfeldt and Porse [33]. Conditioned medium derived from L929 cells grown in RPMI with 10% FBS was used as a source of macrophage colony-stimulating factor (M-CSF), with routine batch monitoring using a mouse M-CSF DuoSet ELISA kit (R&D Systems); batch-to-batch variation was low, approximately 500 pg/ml M-CSF. Eight to 12 week old C57BL/6 mice were euthanised and bone marrow homogenates from hind leg femurs and tibiae were incubated in petri dishes in RPMI (supplemented with 10% FBS and 100 units/ml penicillin and 0.1 mg/ml streptomycin), with 20% conditioned L929 medium for six days at 37 ^o^C, 5% CO_2_, adding fresh medium after three days. After washing the dishes to remove non-adherent cells, the differentiated macrophage population was re-plated as desired. Adherent macrophages were infected with stationary phase promastigotes at a 10:1 parasite:macrophage ratio, and infected macrophages were incubated at 32 ^o^C, 5% CO_2_. After initial infection, extracellular parasites were removed by washing five times with serum-free RPMI, and throughout experiments three more washes were applied every day. For experiments on infection and intracellular growth, infection was for four hours prior to removal of parasites, followed by variable incubation times. For determination of AmB IC_50_, a 24 hour infection was used, prior to addition of variable drug concentrations; infected macrophages were then incubated for a further 48 hours. Infection levels were determined by Giemsa staining and counting (counting at least 100 macrophages per sample). For IC_50_ determination, the ratio of total parasites to total macrophages was used, with IC_50_ values calculated using the R package drc, as described above, applying two-tailed student’s *t* tests of significance. For qRT-PCR experiments, four hours infection and 72 hours subsequent incubation was used, and RNA was extracted using the Nucleospin RNA kit according to manufacturer’s instructions except that initial lysis was carried out by washing cells three times with warm PBS, followed by application of lysis buffer directly to wells. All biological replicates were performed using macrophages derived from separate mice, and separate stationary phase promastigote cultures.

For assessment of infectivity *in vivo*, 2 x 10^6^ stationary phase promastigotes, resuspended in sterile PBS, were injected into the footpads of 8–12 week old female BALB/c mice. Swelling was measured with footpad callipers, taking an average of three independent measurements. For initial infection of wild-type and AmBRC/cl3 promastigotes, parasites were recovered from draining popliteal lymph nodes, four weeks after infection, by physically disrupting lymph nodes and forcing these through a 100 μm cell sieve (EASYstrainer, Grenier Bio-One). Homogenates were incubated in HOMEM supplemented with 10% FBS, 200 units/ml penicillin/0.2 mg/ml streptomycin, and 50 μg/ml gentamycin for several days at 25 ^o^C, by which time proliferating promastigotes were apparent. These recovered parasites were then used to repeat infections (four passages since recovery) in new mice, five per parasite line.

### Ethics statement

All animal experiments were performed in accordance with the Animals (Scientific Procedures) Act 1986 and the University of Glasgow care and maintenance guidelines. All animal protocols and procedures were approved by The Home Office of the UK government and the University of Glasgow Ethics Committee under Project license PCF371688.

## Results

### Selection of four novel AmB-resistant *L*. *mexicana* lines

In order to identify mechanisms of AmB resistance, we selected resistance independently in four *L*. *mexicana* promastigote lines: AmBRA, AmBRB, AmBRC and AmBRD. After culturing cells continuously in stepwise-increasing concentrations of the drug for around 280 days, variable levels of resistance were obtained, ranging from 2.5-fold in AmBRD up to approximately tenfold in AmBRB. Sensitivity to AmB was determined in three clonal populations derived from each line (S1 Table). No significant differences in sensitivity were observed between clones from the same line, hence we proceeded to characterise a single clone per line. In comparison to an AmB IC_50_ value of 58.5 nM in wild-type parasites, IC_50_ values in resistant lines were 427 nM (AmBRA/cl1), 492 nM (AmBRB/cl2), 231 nM (AmBRC/cl3) and 153 nM (AmBRD/cl2), with resistance retained after 15 passages in the absence of drug (Table 1). Only AmBRA/cl1 showed evidence of a mild growth defect in comparison to wild-type parasites (S1 Fig).

**Table 1:** Sensitivity of lines selected for AmB resistance to other antileishmanial agents.

	Wild-type	AmBRA/cl1	AmBRB/cl2	AmBRC/cl3	AmBRD/cl2
**Amphotericin B**	0.0585 ± 0.00314	**0.427 ± 0.0225** **(P = 5.62 x 10**^**−8**^**)**	**0.492 ± 0.0496** **(P = 2.28 x 10**^**−6**^**)**	**0.231 ± 0.0282** **(P = 1.88 x 10**^**−5**^**)**	**0.153 ± 0.0370** **(P = 0.00223)**
**Amphotericin B (15 passages)**	0.0541 ± 0.0135	**0.437 ± 0.0456** **(P = 1.53 x 10**^**−4**^**)**	**0.574 ± 0.101** **(P = 9.06 x 10**^**−4**^**)**	**0.228 ± 0.0136** **(P = 9.67 x 10**^**−5**^**)**	**0.124 ± 0.0242** **(P = 0.0121)**
**Potassium antimonyl tartrate**	105 ± 14.1	94.4 ± 14.2 (P = 0.323)	**65.9 ± 2.51** **(P = 0.00152)**	85.2 ± 17.4 (P = 0.123)	146 ± 48.4 (P = 0.155)
**Miltefosine**	41.6 ± 9.87	**10.7 ± 1.01** **(P = 7.87 x 10**^**−4**^**)**	**96.1 ± 2.35** **(P = 2.60 x 10**^**−4**^**)**	**22.0 ± 2.57** **(P = 0.0106)**	29.5 ± 5.83 (P = 0.0783)
**Paromomycin**	57.5 ± 17.6	**13.9 ± 1.45** **(P = 0.00262)**	60.3 ± 8.30 (P = 0.785)	**132 ± 45.1** **(P = 0.0219)**	87.9 ± 33.5 (P = 0.161)
**Pentamidine**	2.87 ± 0.0648	**0.613 ± 0.0191** **(P = 7.57 x 10**^**−10**^**)**	**0.313 ± 0.0284** **(P = 4.73 x 10**^**−10**^**)**	**0.702 ± 0.0304** **(P = 1.36 x 10**^**−9**^**)**	**0.694 ± 0.0292** **(P = 1.28 x 10**^**−9**^**)**
**Glucose oxidase**	2.39 ± 0.0320	**1.78 ± 0.0877** **(P = 1.30 x 10**^**−5**^**)**	2.84 ± 1.06 (P = 0.423)	2.89 ± 1.11 (P = 0.402)	2.19 ± 0.180 (P = 0.0783)
**Menadione**	5.68 ± 0.685	4.81 ± 0.789 (P = 0.148)	**4.08 ± 0.224** **(P = 0.00443)**	4.94 ± 0.698 (P = 0.181)	6.34 ± 0.710 (P = 0.229)
**Methylene blue**	5.67 ± 1.31	**0.186 ± 0.0425** **(P = 1.62 x 10**^**−4**^**)**	**0.301 ± 0.0592** **(P = 1.82 x 10**^**−4**^**)**	**1.57 ± 1.29** **(P = 0.00437)**	**1.18 ± 0.243** **(P = 5.26 x 10**^**−4**^**)**

Sensitivity of wild-type (WT) and AmB-resistant lines was determined by Alamar Blue assay. IC_50_ values are given in μM, with the exception of glucose oxidase, which is in mU/ml. Mean values of four independent biological replicates are shown ± standard deviation. P values determined by two-tailed Student’s *t*-test are shown, with values considered statistically significant (P < 0.05) in bold.

We tested sensitivity of each line to a range of antileishmanial drugs (Table 1). Changes were for the most part not conserved between lines; for example, miltefosine hypersensitivity was found in AmBRA/cl1 and AmBRC/cl3, whilst AmBRB/cl2 was 2.3-fold resistant. However, all lines were hypersensitive to pentamidine, consistent with previous observations [18,21]. We also examined sensitivity to oxidative stress-inducing agents, as hypersensitivity has previously been reported in AmB-resistant *Leishmania* [21]. Substantial changes in sensitivity to both glucose oxidase (an extracellular source of H_2_O_2_) and menadione (a source of intracellular superoxide) were not observed, but all lines were hypersensitive to methylene blue (30-fold in AmBRA/cl1), which creates stress through oxidation of NADPH to NADP^+^ [34].

### Two types of changes to sterol composition are observed in resistant lines

**Fig 1:**
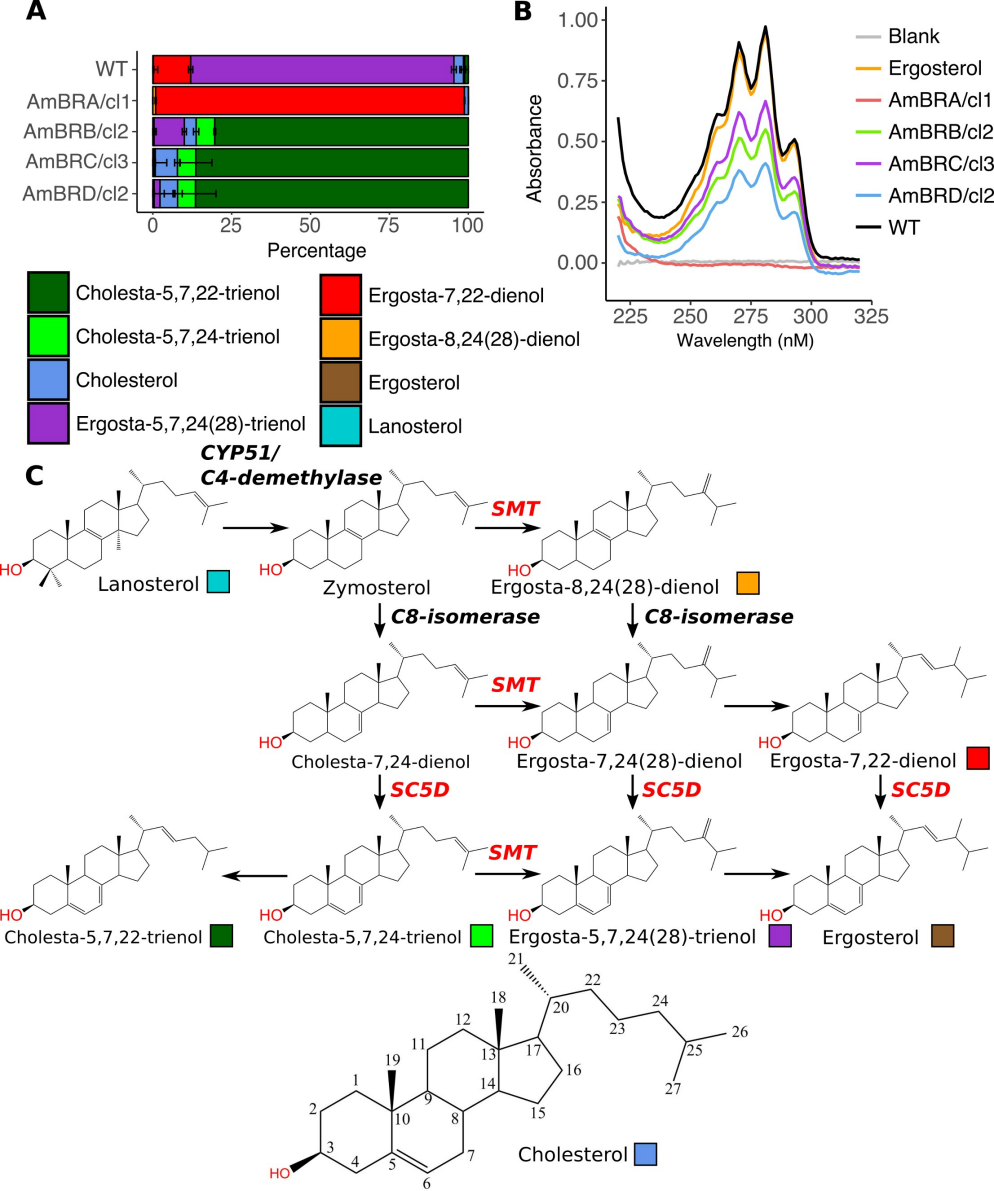
Changes to sterol composition in AmB-resistant parasites. A) Percentage sterol composition, as determined by GC-MS. Error bars represent standard deviation of the mean, n = 3. B) UV absorbance spectra of sterol extracts. Ergosterol dissolved in *n* heptane (0.05 mg/ml) is used as a standard, and *n*-heptane as a blank. Representative of three independent biological replicates. C) Sterol biosynthesis pathway in *Leishmania*, showing the structures of sterols identified in wild-type and AmB-resistant parasites. Coloured boxes relate to the colour key for sterols in panel A. Cholesterol is shown with the carbons numbered as referred to in the text.

As altered sterol composition was previously observed in AmB resistance, we examined changes by gas chromatography-mass spectrometry (GC-MS) (Fig 1A & 1C, S2 & S3 Tables). The major sterol (83.4%) of wild-type parasites was an isomer of ergosterol; however, as its retention time did not match to the ergosterol standard used, we identified it as ergosta-5,7,24(28)-trienol, based on previous literature [35,36]. Ergosta-7,22-dienol was also present at 11.8% of total sterol content. In all resistant lines, ergosta-5,7,24(28)-trienol was lost or greatly depleted; however, two types of profile emerged in resistant lines. In AmBRA/cl1, almost all sterol (97.9%) consisted of ergosta-7,22-dienol. This is C24-methylated but lacks ergosterol-like 5(6)-7(8) double bond conjugation. By contrast, AmBRB/cl2, AmBRC/cl3 and AmBRD/cl2 all exhibited accumulation of sterols lacking C24-methylation, particularly cholesta-5,7,22-trienol (80.4%, 86.4% and 86.5%, respectively). Double bond conjugation in the ergosterol ring structure results in a distinctive UV absorbance spectrum [37]. Spectroscopy of sterol extracts indicated loss of this conjugation in AmBRA/cl1, but retention in the other three lines, further supporting evidence from GC-MS (Fig 1B).

### Whole genome sequencing identifies putative resistance-associated genetic changes

Whole genome sequencing (WGS) demonstrated extensive chromosomal copy number variation (CNV), but with little conservation of karyotypic changes across four lines, suggesting such heterogeneity may be stochastic (S2 Fig). Non-synonymous mutations in sterol biosynthesis genes were limited to two enzymes. In AmBRA/cl1, a unique homozygous mutation (G415C) encoded a G139R amino acid substitution in sterol C5-desaturase (SC5D, *LmxM*.*23*.*1300*), which is required for generation of sterol 5(6)-7(8) double bond conjugation (which, notably, is lost in this line). Analysis of SNPs in this region demonstrated evidence of large-scale loss of heterozygosity, leading to the homozygous variant (S3 Fig). Alignment to SC5D orthologues suggested proximity of G139R to conserved His residues implicated in catalysis (S4 Fig) [38].

In AmBRB/cl2, AmBRC/cl3 and AmBRD/cl2, changes were observed in SMT, which in *L*. *mexicana* is encoded by two tandemly arrayed gene copies, *LmxM*.*36*.*2380* and *LmxM*.*36*.*2390*. These gene copies differ only by a single nucleotide at position 391 in the reference genome (G in *LmxM*.*36*.*2380*, A in *LmxM*.*36*.*2390*), but in our data, a G961A substitution was also identified in wild-type *LmxM*.*36*.*2390*. Both substitutions result in Val to Ile coding changes. The high homology of coding sequences may impair correct assignment of SNPs to different gene copies. Therefore, in Table 2, we examine genotypes taking both copies together. This shows that in AmBRB/cl2 and AmBRC/cl3, A residues are found at both positions (A391/A961), whereas in AmBRD/cl2, a G391/A961 genotype is observed. A secondary homozygous SNP, T215G, was observed uniquely in AmBRC/cl3, encoding a F72C substitution. Alignment to the yeast SMT orthologue shows that this falls within a putative sterol binding site (S5 Fig) [39], and genotyping of cryogenically preserved intermediate stages of selection demonstrated that this mutation occurred independently, subsequent to the changes at positions 391 and 961 (S6 Fig).

**Table 2:** Genotypes of sterol biosynthesis genes in wild-type and resistant lines.

	SC5D	SMT		
	415 (139)	215 (72)	391 (131)	961 (321)
**WT**	G/G (G/G)	T/T (F/F)	G/A (V/I)	G/A (V/I)
**AmBRA/cl1**	C/C (R/R)	T/T (F/F)	G/A (V/I)	G/A (V/I)
**AmBRB/cl2**	G/G (G/G)	T/T (F/F)	A/A (I/I)	A/A (I/I)
**AmBRC/cl3**	G/G (G/G)	G/G (C/C)	A/A (I/I)	A/A (I/I)
**AmBRD/cl2**	G/G (G/G)	T/T (F/F)	G/G (V/V)	A/A (I/I)

Where variants were detected in sterol biosynthesis genes, genotypes at these loci are shown, along with the coded amino acids in brackets. SMT genotypes combine both SMT copies, *LmxM*.*36*.*2380* and *LmxM*.*36*.*2390*, due to the difficulty of discriminating between these gene copies by WGS.

**Fig 2:**
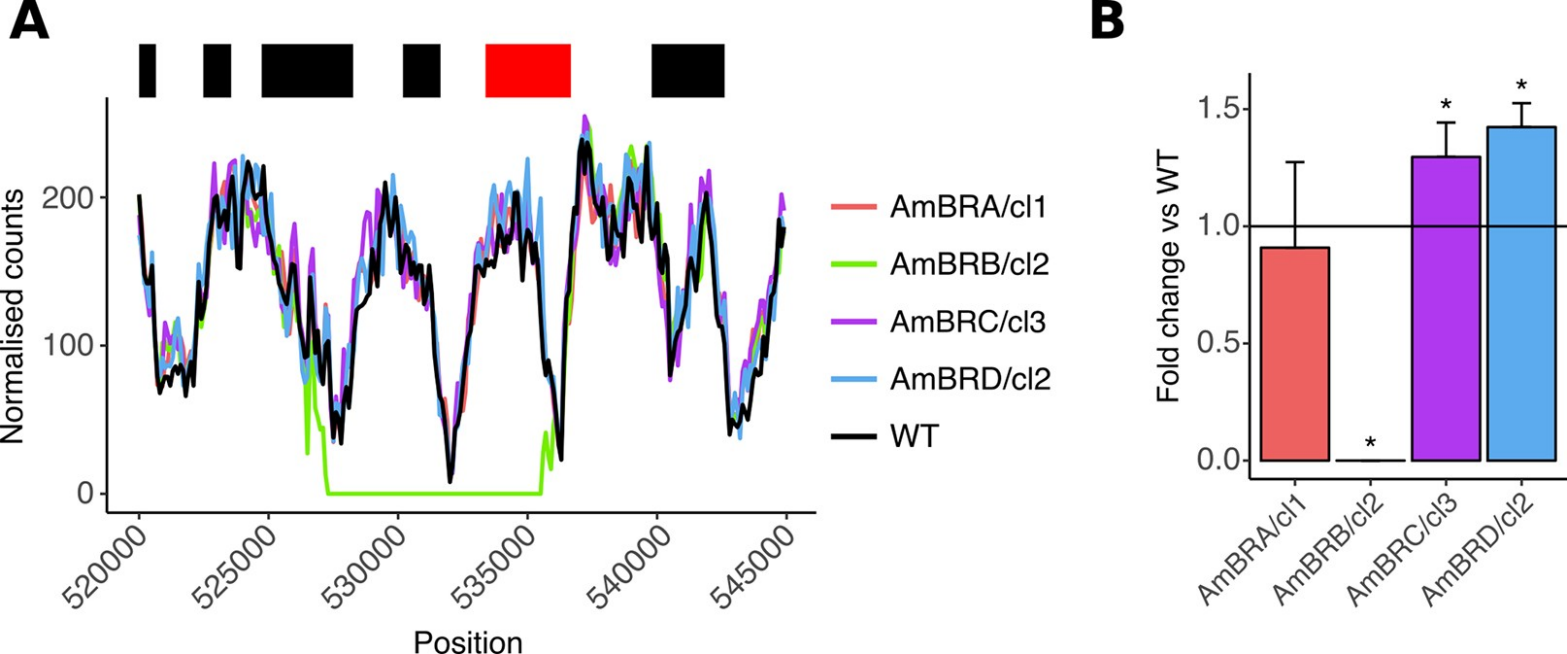
Deletion of the miltefosine transporter. A) Genome coverage at the transporter locus. Normalised mapped read count for 100 bp windows is plotted against genomic position on chromosome 13 (normalisation was achieved by adjusting counts by the ratio between total mapped read counts for chromosome 13 for that line compared to wild-type). The top banner shows local read positions as black boxes, with the exception of the miltefosine transporter (*LmxM*.*13*.*1530*), which is in red. B) Miltefosine transporter expression, as determined by qRT-PCR. Asterisks denote statistically significant differences (P < 0.05) in δCt from wild-type (P values for AmBRB/cl2, AmBRC/cl3 and AmBRD/cl2 are 7.32 x 10^−6^, 0.0165 and 0.00105, respectively), n = 3. See Methods for the statistical approach used.

Per-gene coverage analysis also revealed deletion of an 8 kb region (Fig 2A) in AmBRB/cl2, encoding *LmxM*.*13*.*1530* and *LmxM*.*13*.*1540*, the former of which encodes the miltefosine transporter, which has previously been implicated in miltefosine resistance [7,31,40,41], with some SNPs implicated in miltefosine-AmB cross-resistance [20]. Loss of expression of this gene was confirmed by qPCR (Fig 2B) and genotyping of intermediate stages revealed loss of this gene prior to the changes in SMT in this line. As for SC5D, homozygous transporter deletion was associated with loss of heterozygosity on the parental chromosome (S3 Fig).

### RNA-seq reveals altered SMT transcript expression

In order to determine changes to transcript abundance associated with drug resistance, we performed RNA-seq transcriptomics analysis. To capture maximal biological variability, we used three clones from each resistant line (and three separate flasks of wild-type parasites), treating these as biological replicates. In each line, very high numbers of differentially expressed genes were reported, ranging from 13% to 41% of all detected coding sequences (S1 Data); however, most of these were moderate (less than twofold) fold changes (S7 Fig). Altered chromosomal ploidy exerted a clear influence on RNA expression levels (S8 Fig).

**Fig 3:**
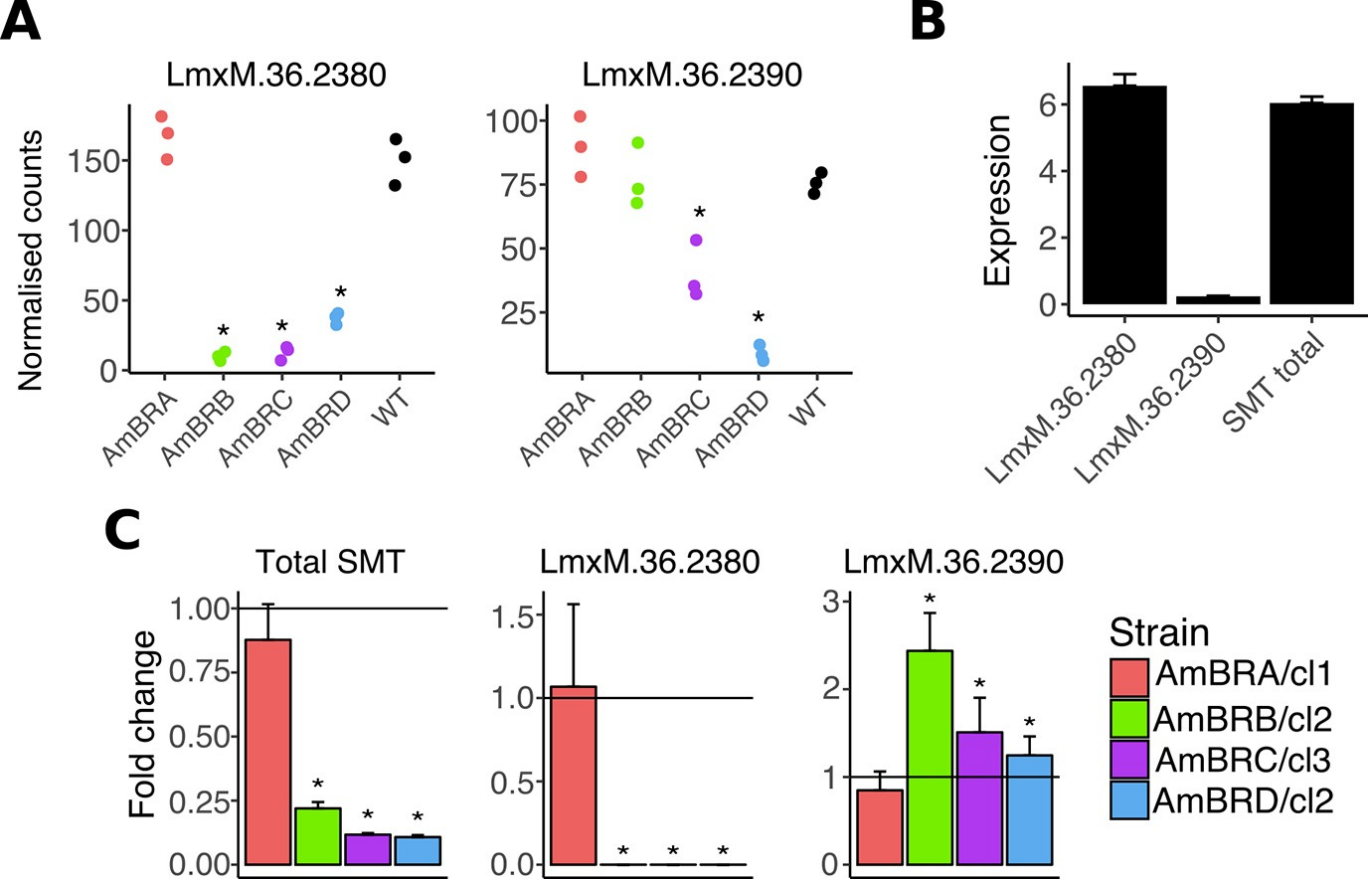
Changes to SMT expression. A) Expression of SMT genes *LmxM*.*36*.*2380* and *LmxM*.*36*.*2390*, expressed as normalised counts for individual clones for resistant lines (three independent biological replicates for wild-type). Asterisks represent corrected P values < 0.05. See Supplementary data for full statistical information. B) Wild-type promastigote expression of SMT genes, expressed as fold-expression over GAPDH. Initial Ct values were normalised to standard curves of genomic DNA to allow direct comparison of different qPCR targets (overall SMT values were altered by a factor of two to account for two gene copies), followed by division by similarly normalised values for GAPDH. Note that there is no significant difference (P > 0.05, two-tailed student’s *t-*test) in values for *LmxM*.*36*.*2380* and total SMT expression. C) Expression of SMT genes in AmB-resistant lines, as determined by qRT-PCR. Asterisks denote statistically significant differences (P < 0.05) in δCt from wild-type, n = 3. P values for statistical changes were as follows for AmBRB/cl2, AmBRC/cl3 and AmBRD/cl2, respectively: for overall SMT, 4.68 x 10^−6^, 9.70 x 10^−6^ and 1.58 x 10^−6^; for *LmxM*.*36*.*2380*, 4.05 x 10^−5^, 2.21 x 10^−4^, and 0.00318; for *LmxM*.*36*.*2390*, 4.71 x 10^−4^, 0.0228 and 0.0430. See Methods for the statistical approach used.

Two of the genes demonstrating the largest fold changes in expression were those encoding SMT, *LmxM*.*36*.*2380* and *LmxM*.*36*.*2390* (Fig 3A). Decreases in *LmxM*.*36*.*2380* were observed in AmBRB, AmBRC and AmBRD, with *LmxM*.*36*.*2390* also reduced in AmBRC and AmBRD. Given the similarity of coding sequences, and resulting difficulties in distinguishing through RNA-seq, we exploited differences in 3’-UTR sequences to design qPCR primers for quantification of individual gene copies, after correcting incomplete assembly in the intergenic region of the reference genome by Sanger sequencing (S1 Text). This allowed specific amplification and sequencing of each coding sequence, confirming the presence of G residues at positions 391 and 961 for *LmxM*.*36*.*2380*, and A residues at these positions in *LmxM*.*36*.*2390*. qRT-PCR revealed dominance of *LmxM*.*36*.*2380* expression over *LmxM*.*36*.*2390* in wild-type promastigotes (28-fold higher) (Fig 3B). qRT-PCR targeting a region of coding sequence that did not differ between gene copies confirmed an overall reduction in SMT expression in these lines, 0.22-fold (AmBRB/cl2), 0.12-fold (AmBRC/cl3) and 0.11-fold (AmBRD/cl2) that of wild-type (with AmBRA/cl1 unchanged) (Fig 3C). However, this was transcript specific: reduced overall expression was driven by total loss of the dominant copy, *LmxM*.*36*.*2380*, with little change in *LmxM*.*36*.*2390*. Comparison of RNA-seq and qRT-PCR measurements of SMT expression show discrepancies between the two methods with respect to relative abundance of the two gene copies. This is likely an effect of mapping reads incorrectly in the RNA-seq data due to the high similarity between sequences, as well as either issues with the reference genome or structural rearrangements between lines. Hence the qRT-PCR assay, which targets regions specific to each 3’-UTR, is the more reliable estimate of expression in this context.

### Functional validation of the role of resistance-associated changes

**Fig 4:**
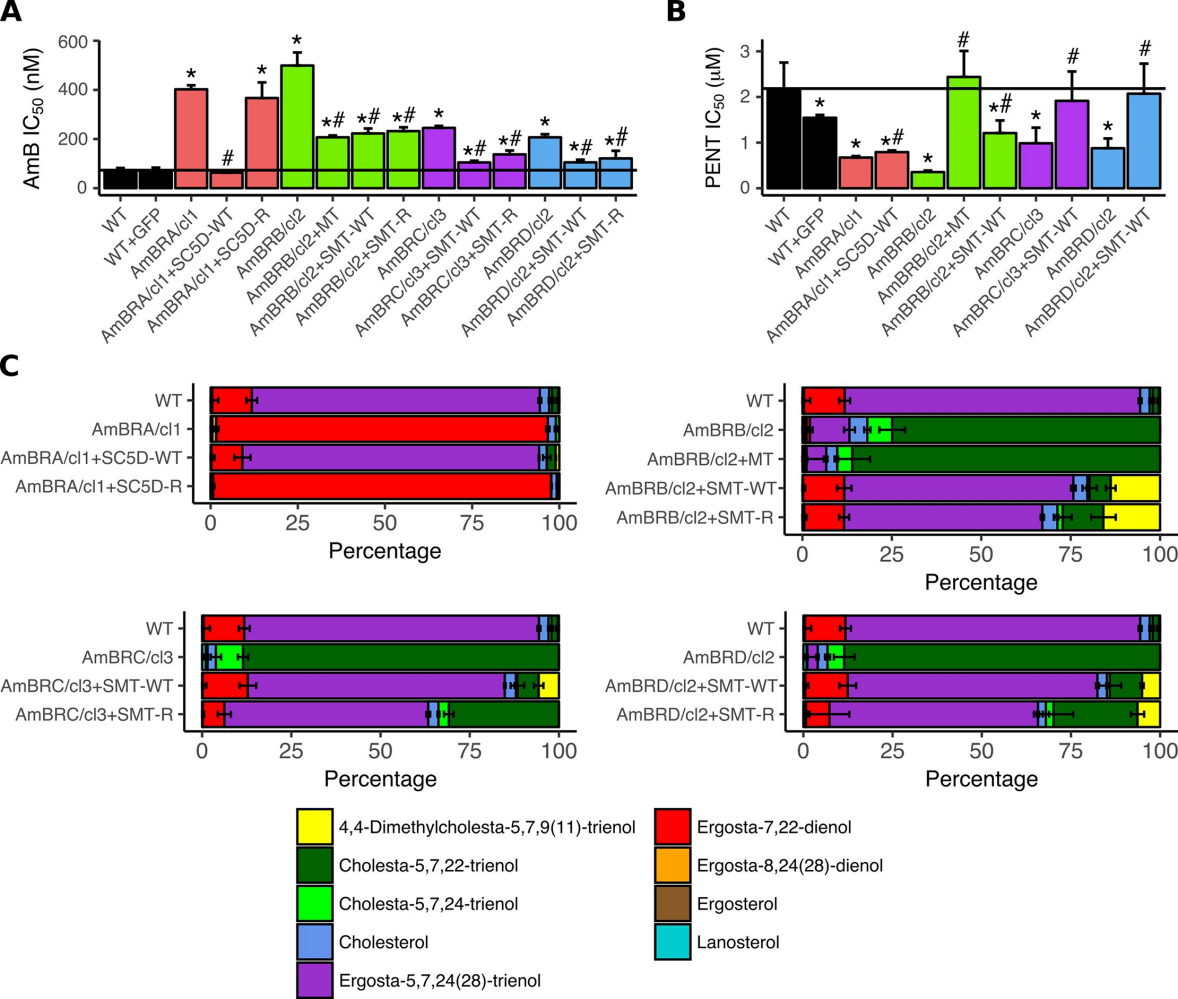
Effects of ectopic expression of candidate AmB resistance-associated genes. A) AmB sensitivity. Mean values are shown for IC_50_ with error bars denoting standard deviation (n = 4, except for AmBRB/cl2 and AmBRD/cl2 (n = 6), and AmBRB/cl2 + MT, AmBRB/cl2 + SMT WT and AmBRD/cl2 + SMT WT (n = 8)). Asterisks represent significant differences (P < 0.05), compared to wild-type, hashes between ectopic overexpression lines and their parental AmB-resistant lines. The black vertical bar denotes wild-type sensitivity. See S5 Table for full data, including individual P values. B) Pentamidine (PENT) sensitivity. As for panel (A), with n = 5 for all lines. C) Percentage sterol composition, as determined by GC-MS. Error bars represent standard deviation of the mean, n = 3. MT: Miltefosine transporter.

To verify whether changes to SC5D, SMT and the miltefosine transporter were causative of resistance we reintroduced wild-type copies of these genes (*LmxM*.*36*.*2380* for wild-type SMT) to AmB-resistant lines as well as the versions of SC5D and SMT derived from the resistant lines themselves. Reintroduction of wild-type SC5D into AmBRA/cl1 caused total restoration of AmB sensitivity while the G139R mutant version had no effect (Fig 4A, S5 Table). By contrast, in all cases, reintroduction of SMT caused only partial restoration of AmB sensitivity. Ectopic expression of resistant line-derived SMT copies did not differentially affect AmB sensitivity in comparison to reintroduction of the wild-type coding sequence *LmxM*.*36*.*2380* (P = 0.471 for AmBRB/cl2, P = 0.263 for AmBRD/cl2), with the exception of AmBRC/cl3, for which ectopic overexpression of AmBRC/cl3-derived SMT caused significantly less restoration of AmB sensitivity than the wild-type copy (P = 0.0161), possibly as a result of the secondary F72C coding sequence substitution in this line. Reintroduction of miltefosine transporter expression also resulted in partial restoration of AmB sensitivity. GFP expressed in wild-type cells, introduced to control for the presence of the vector and the selection marker G418, did not influence AmB sensitivity.

GC-MS sterol analysis on transfected lines (Fig 4C, S4 Table) showed that reintroduction of SC5D and SMT, but not the miltefosine transporter, resulted in restoration of wild-type sterol composition, with ergosta-5,7,24(28)-trienol becoming the dominant sterol. In the case of SMT overexpression, however, as with reversion of resistance, this was incomplete, with C24-methylated (ergosta-type) sterols still reduced relative to wild-type and C24-unmethylated (cholesta-type) higher. Furthermore, a novel sterol not detectable in any untransfected lines, 4,4-dimethylcholesta-5,7,9(11)-trienol, formed 5–16% of total sterol in SMT-transfected lines. Given that SMT expression in these lines was five- to 16-fold that of wild-type (S9 Fig), this novel sterol could be a result of overexpression of this enzyme, allowing it to generate products through alternative reactions. Moreover, accumulation of this additional sterol could explain the failure of SMT overexpression to fully restore wild-type sensitivity. Only in AmBRC/cl3 were substantial differences noted between wild-type and resistant line-derived copies, with the novel sterol 4,4-dimethylcholesta-5,7,9(11)-trienol not detected and C24-unmethylated cholesta-5,7,22-trienol substantially higher in parasites transfected with AmBRC/cl3-derived (F72C) SMT.

Finally, as pentamidine hypersensitivity was detected in all AmB-lines, we tested the effect of gene reintroduction (Fig 4B, S5 Table). All overexpressed genes caused significant increases in pentamidine IC_50_, albeit marginal for SC5D. Miltefosine transporter overexpression in AmBRB/cl2 caused the largest rise (6.9-fold), whereas SMT reintroduction also caused a two- to threefold increase in IC_50_ in all cases. Hence, pentamidine hypersensitivity can result from both sterol dependent and independent routes.

### Changes in SMT expression are associated with genomic copy number variants

**Fig 5:**
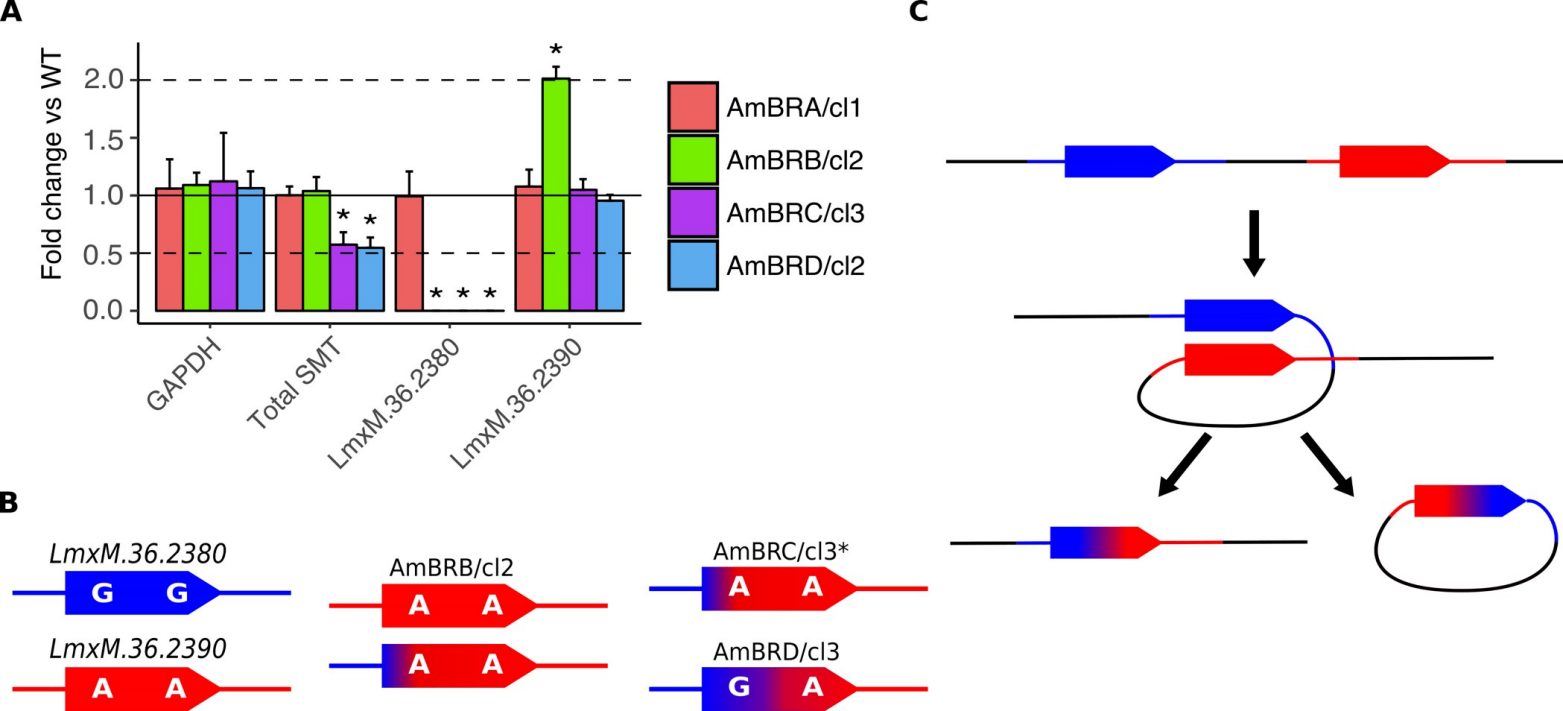
Genomic changes at the SMT locus. A) Copy number variations in GAPDH (*LmxM*.*36*.*2350*) and SMT genes (*LmxM*.*36*.*2380* and *LmxM*.*36*.*2390*) as determined by qPCR. Fold changes are calculated as 2^-δCt^ between each line and wild-type, with 5 ng genomic DNA loaded per sample. Asterisks denote statistically significant differences (P < 0.05) in δCt from wild-type, n = 3. Dotted lines denote a halving or doubling of gene copy number. P values for statistically significant changes are as follows: for total SMT, values are 0.00302 and 0,00256 for AmBRC/cl3 and AmBRD/cl2, respectively; for *LmxM*.*36*.*2380*, values are 1.52 x 10^−5^, 5.78 x 10^−6^ and 4.39 x 10^−4^ for AmBRB/cl2, AmBRC/cl3 and AmBRD/cl2, respectively; for *LmxM*.*36*.*2390*, the value for AmBRB/cl2 is 0.00105. See Methods for the statistical approach used. B) Transcript sequences present in wild type (left column) and resistant lines. C) A model for deletion of the intergenic region and formation of a chimaeric SMT gene copy. Homologous recombination leads to generation of an extrachromosomal circular fragment, which is subsequently lost during replication.

We then probed the genomic changes driving diminished expression. qPCR on genomic DNA revealed a halving of SMT copy number in AmBRC/cl3 and AmBRD/cl2, with wild-type copy number retained in AmBRA/cl1 and AmBRB/cl2 (Fig 5A). This was associated in AmBRC/cl3 and AmBRD/cl2 with loss of the *LmxM*.*36*.*2380* 3’-UTR but retention of the *LmxM*.*36*.*2390* 3’-UTR. In AmBRB/cl2, whilst overall copy number was retained, there was a similar loss of the *LmxM*.*36*.*2380* 3’-UTR; however, the *LmxM*.*36*.*2390* 3’-UTR was doubled. This was confirmed by PCR of coding sequences using 3’-UTR-specific reverse primers (S10 Fig). Moreover, PCR amplification of the intergenic region between SMT gene copies yielded a product only from wild-type and AmBRA/cl1 genomic DNA, suggesting that tandemly arranged gene copies were no longer present in the other lines, even though overall SMT copy number is maintained in AmBRB/cl2 (S10 Fig). Having corrected the SMT intergenic region in the reference genome, we realigned WGS and RNA-seq data to this corrected reference, confirming loss of this region in AmBRC/cl3 and AmBRD/cl2 (S11 & S12 Figs). Interestingly, this included loss of the 5’-UTR of *LmxM*.*36*.*2390*, whilst the 5’-UTR of *LmxM*.*36*.*2380* was retained. Given that only the *LmxM*.*36*.*2390* 3’-UTR is found in these lines, this implies that the resulting SMT copy is actually a chimeric fusion of the two wild-type SMT copies, with the interceding intergenic region lost (Fig 5B). By contrast, in AmBRB/cl2, only part of the intergenic region is lost (within the 3’-UTR of *LmxM*.*36*.*2380*), whereas coverage of both 5’-UTRs is retained. This suggests that in AmBRB/cl2, two types of SMT are present, each with a separate 5’-UTR but both with the same (*LmxM*.*36*.*2390*-derived) 3’-UTR (Fig 5B).

For AmBRC/cl3 and AmBRD/cl2, we can propose a model to explain the above observations. Previously, homologous recombination-mediated deletion has been proposed in *Leishmania* [42,43]. Here, homologous recombination arising during double strand break repair would involve looping of intergenic DNA (2.9 kb) and alignment of the two SMT gene copies (99.8% homology), resulting in excision and subsequent loss of the looped region (Fig 5C). The resulting transcript inherits the 5’-UTR from *LmxM*.*36*.*2380* and the 3’-UTR from *LmxM*.*36*.*2390*. Although the genotypes differ between AmBRC/cl3 and AmBRD/cl2, this could result from differential repair of the two mismatches at positions 391 and 961, or be dependent on the location of the initial break or the degree of resection. This model cannot explain, however, the conservation of copy number in AmBRB/cl2.

### SIDER1 repetitive elements are associated with multigenic amplification in AmB resistance

**Fig 6:**
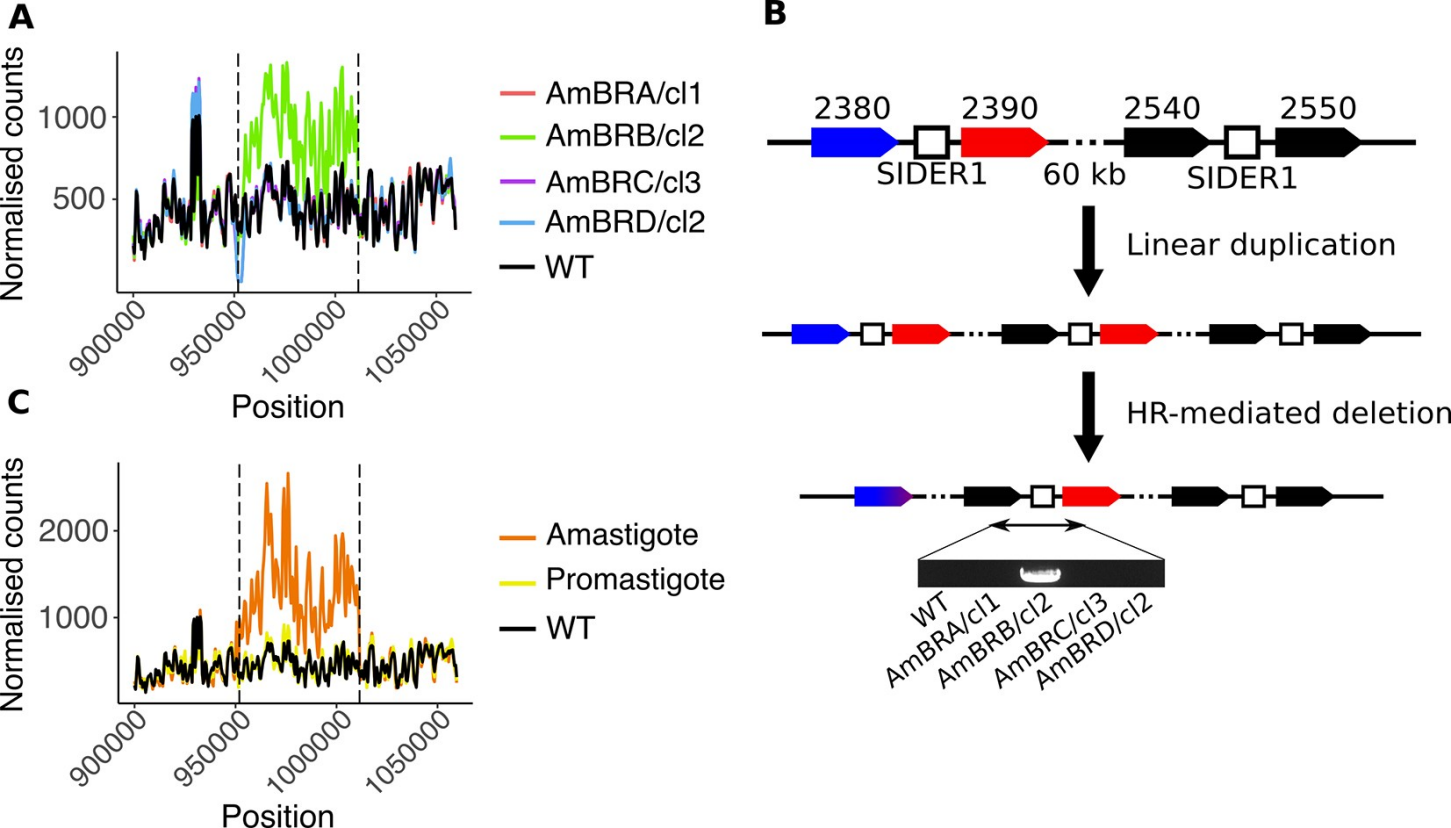
Structural changes associated with SIDER1-mediated amplification. A) Genome coverage of the region around the SMT locus. Normalised mapped read count for 500 bp windows is plotted against genomic position on chromosome 20 (normalisation was achieved by adjusting counts by the ratio between total mapped read counts for chromosome 20 for that line compared to wild-type). Vertical dotted black lines denote the mid-point position of the two tandem SIDER1 elements. B) A model for structural changes in AmBRB/cl2. Initial SIDER1-mediated linear duplication of a ~60 kb region is followed by homologous recombination-mediated deletion as depicted in Fig 5D. Note that whether these steps happen sequentially or simultaneously cannot be determined. Amplification leads to proximity between *LmxM*.*36*.*2540* and an SMT gene copy, which is only detectable by PCR in AmBRB/cl2, as shown here (for full gel image see S10 Fig). C) Genome coverage of the region around the SMT locus in a previously selected AmB-resistant amastigote line. See panel (A) for details. Coverage data for a promastigote line derived from the same parent that did not exhibit amplification, as well as the wild-type line used in this study, are included for comparison.

Sequencing the intergenic region between SMT gene copies revealed a region of sequence that shared strong homology to other regions within chromosome 20, including a 450 bp region of sequence between *LmxM*.*36*.*2540* and *LmxM*.*36*.*2550* (approximately 50 kb away from the SMT locus) with ~98% identity to this sequence (five gaps and three nucleotide differences). Homologous syntenic regions in *L*. *major* and *L*. *infantum* that have previously been identified as small interspersed degenerate retrotransposon (SIDER) 1 elements (Supplementary text 2) [44]. SIDER elements promote copy number instability, including amplifications [43,44]. Analysis of per-gene coverage in the WGS data clearly showed a duplication event spanning 16 genes from *LmxM*.*36*.*2390* to *LmxM*.*36*.*2540* in AmBRB/cl2, its boundaries coinciding with the sites of these putative SIDER1 elements (Fig 6A). qPCR confirmed evidence of gene duplication in this line (S13 Fig).

Tandem repeat-mediated amplification in *Leishmania* has been described previously [43]. Intrachromosomal duplication is mediated by misalignment of sister chromatids, resulting in proximity between the last gene in the amplicon (here, *LmxM*.*36*.*2540*) and the first (*LmxM*.*36*.*2390*); using PCR primers specific to the two genes, we found that such a junction was indeed present in AmBRB/cl2, but not wild-type DNA (Fig 6B). We propose that the genomic structural changes in AmBRB/cl2 arise through linear duplication, accompanied or followed by deletion of the inter-SMT region through a similar homologous recombination mechanism as described for AmBRC/cl3 and AmBRD/cl2 (Fig 6B). This results in destruction of the inter-SMT SIDER1 element (as evidenced by loss of the intergenic region, S10 Fig), stabilising the lesion.

If these repetitive elements do contribute to instability, they should recur in other lines. We obtained and sequenced a previously described AmB-resistant *L*. *mexicana* axenic amastigote line alongside a promastigote line derived from the same parent [19]. Whilst the promastigote line did not differ from our wild-type parasites at this locus, the resistant amastigotes indicated a three-fold amplification of the same region we found in AmBRB/cl2 (Fig 6C). A homozygous G to A mutation in *LmxM*.*23*.*1300* (SC5D) led to substitution of W242 to a stop codon, an event that is likely to have a strong effect on gene function and therefore complicates our understanding of resistance in this line.

### Infectivity, AmB resistance and SMT expression in intracellular amastigotes

**Fig 7:**
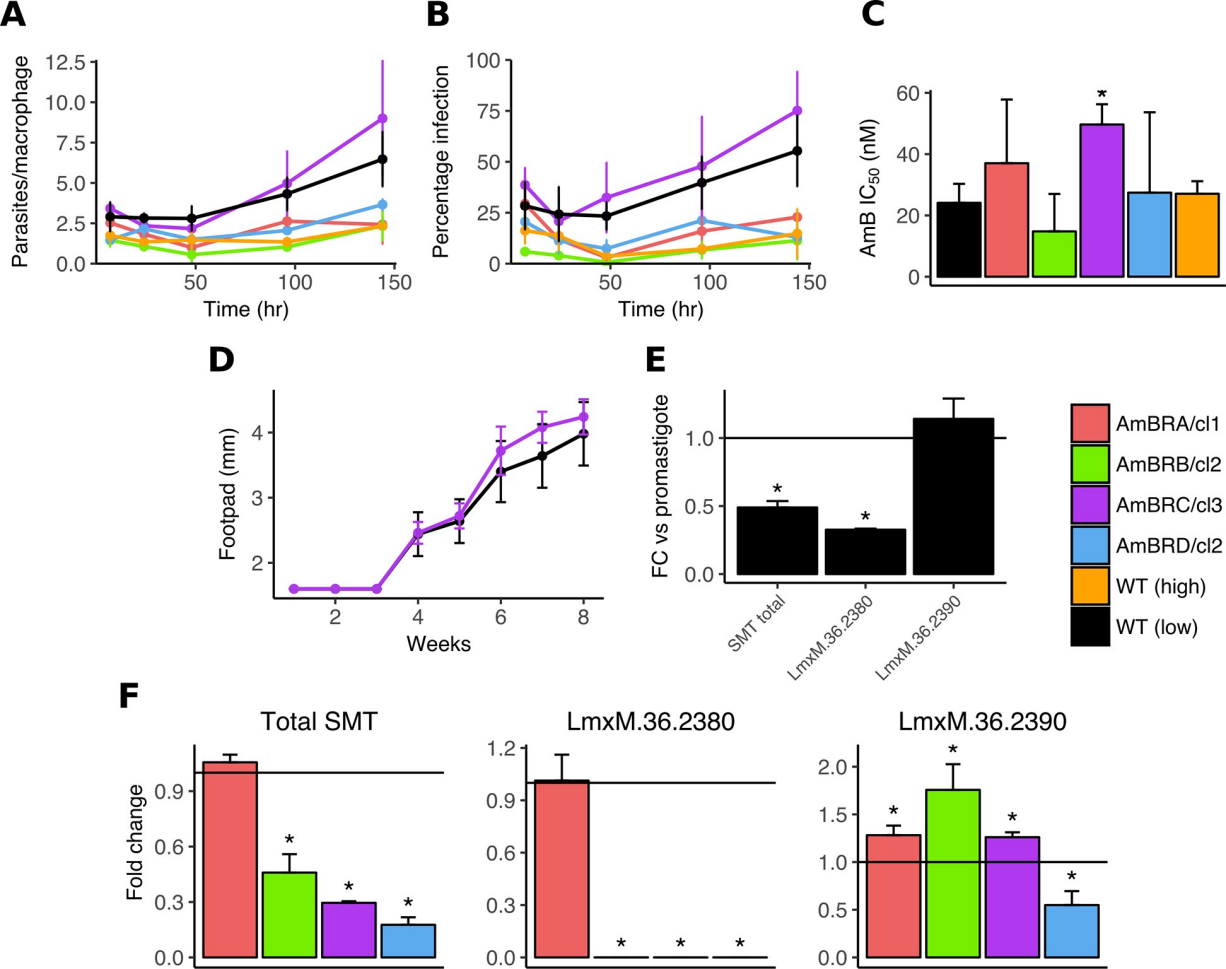
Infectivity of AmB-resistant parasites and SMT gene expression in intracellular amastigotes. A & B) Infectivity and replication of parasites within primary bone marrow-derived murine macrophages. Stationary phase promastigotes were used to infect macrophages for variable lengths of time and infection burdens were quantified as both parasites per infected macrophage (A) and percentage of macrophages infected (B). Error bars denote standard deviation, n = 3. C) AmB sensitivity of intracellular amastigotes. Asterisks significant differences (P < 0.05) in IC_50_ from wild-type, n = 3. D) Infectivity of wild-type and AmBRC/cl3 parasites *in vivo*. Parasites recovered from mouse lymph nodes were brought to stationary phase and injected into mouse footpads. Footpad size was measured over time. The mean of five mice per parasite line is shown, error bars represent standard deviation. E) Fold-change in expression of SMT genes in amastigotes compared to promastigotes in wild-type parasites. Asterisks significant differences (P < 0.05) in δCt between the two life cycle stages (statistically significant P values are 3.00 x 10^−4^ (total SMT) and 1.42 x 10^−5^ (*LmxM*.*36*.*2380*)), n = 3. F) Expression of SMT genes in intracellular amastigotes. RNA was derived from primary macrophages 72 hours after infection with stationary phase promastigotes. Asterisks denote statistically significant differences (P < 0.05) in δCt from wild-type, n = 3. Statistically significant P values are as follows: for total SMT, respective values for AmBRB/cl2, AmBRC/cl3 and AmBRD/cl2 are 0.0270, 0.00106 and 0.00164; for *LmxM*.*36*.*2380*, respective values for AmBRB/cl2, AmBRC/cl3 and AmBRD/cl2 are 0.00164, 6.31 x 10^−6^ and 1.95 x 10^−4^; for *LmxM*.*36*.*2390*, values for AmBRA/cl1, AmBRB/cl2, AmBRC/cl3 and AmBRD/cl2 are 0.0105, 0.0143, 0,0396 and 0,0121. See Methods for the statistical approach used in qRT-PCR assays.

*Leishmania* promastigotes, the form found in infected sandflies, are an experimentally tractable model, but macrophage-resident amastigotes are a closer representation of infection in the mammalian host environment. Hence we examined the ability of resistant lines to infect primary murine macrophages. We used both the parental wild-type line (less than 15 passages since isolation from an infected mouse) and a wild-type line cultured in the absence of drug for more than 50 passages, to control for effects of long-term growth *in vitro*. The ability to infect and replicate within primary macrophages was greatly reduced in this long-passaged line. Similarly, AmBRA/cl1, AmBRB/cl2 and AmBRD/cl2 all had low infection rates (although we cannot distinguish whether this was due to long passage or physiological changes due to resistance selection). By contrast, AmBRC/cl3 showed levels of infection and intracellular replication at or even higher than parental low passage wild-type cells (Fig 7A & 7B). We then examined whether AmB resistance was retained in intracellular amastigotes (Fig 7C). High variability was observed for some lines, likely due in part to the poor infectivity rates even in the absence of drug; but no significant differences were apparent in sensitivity of AmBRA/cl1, AmBRB/cl2 or AMBRD/cl2 when compared to WT. By contrast the IC_50_ of AmBRC/cl3 (49.7 nM ± 6.2 nM) was significantly higher (P = 0.008) than wild-type (24.1 nM ± 6.6 nM).

As AmBRC/cl3 remained highly infectious to macrophages, we wished to determine whether the same was true *in vivo*. To account for differences due to long-term culture *in vitro*, we initially injected wild-type and AmBRC/cl3 parasites into the footpads of BALB/c mice, recovering live parasites from the draining lymph nodes after four weeks. AmB resistance was conserved in isolated AmBRC/cl3 parasites compared to wild-type (IC_50_ 229 nM ± 2.4 nM and 73.7 nM ± 6.7 nM, respectively, P = 9.81 x 10^−9^). Isolates were then used to infect five mice for each line and footpad swelling was monitored over eight weeks as a marker for infection progression; these showed no evidence of loss of infectivity *in vivo* in AmBRC/cl3 relative to wild-type (Fig 7D). We also aimed to determine whether resistance was retained *in vivo*; however the dose used (seven intravenous doses of 1 mg/kg AmB every other day for 12 days) was insufficient to clear either wild-type or resistant parasites, and higher doses proved toxic to the mice.

Whilst poor infectivity and replication may well contribute to the lack of resistance in AmBRA/cl1, AmBRB/cl2 and AmBRD/cl2 intracellular amastigotes, biological factors may play a role. Through qRT-PCR, we demonstrated that overall SMT expression in intracellular amastigotes was 0.49-fold that of promastigotes (Fig 7E), in accordance with previously published RNA-seq data [32]. This change was driven by *LmxM*.*36*.*2380*, which decreased to 0.33-fold that of promastigotes, where *LmxM*.*36*.*2390* was unchanged. Comparison to resistant lines demonstrated that whilst SMT expression overall was still decreased, it was to a lesser degree than in promastigotes, with expression 0.46-fold (AmBRB/cl2), 0.30-fold (AmBRC/cl3) and 0.18-fold (AmBRD/cl2) that of wild-type parasites (Fig 7F), similarly driven by a loss of *LmxM*.*36*.*2380*. Therefore, loss of overall SMT expression is less pronounced in intracellular amastigotes due to changes in the relative abundance of SMT gene copies, which might contribute to narrowing of the discriminatory concentration of drug required to kill wild-type and resistant lines.

## Discussion

AmB has become first line treatment for leishmaniasis in several settings, and a single dosing regimen has been pursued for VL in India in recent years. This approach brings an inherent risk of acquired drug resistance. In order to identify novel genetic mechanisms by which resistance to AmB may arise, we selected four independent resistant *L*. *mexicana* lines in parallel. Whilst numerous genetic and phenotypic changes were observed, these were rarely conserved between multiple resistant lines. This demonstrates the importance of selection of several resistant lines in determining which are essential features of resistance, and which arise stochastically. The only conserved changes in sensitivity to compounds were hypersensitivity to methylene blue and pentamidine. The former could relate to intracellular action of the compound, or simply altered uptake. We show here, however, that pentamidine hypersensitivity arises due to both sterol-dependent and sterol-independent mechanisms. The mechanisms by which these changes lead to AmB resistance merit further investigation. Whilst the mode of action of pentamidine is not entirely clear, changes in lipid distribution and membrane fluidity have previously been implicated in pentamidine resistance *in vitro* [45], providing a possible link between pentamidine sensitivity and the mutations described here. Intriguingly, increased membrane fluidity has been observed in resistance to both pentamidine and AmB [18], despite the apparent negative correlation between sensitivity to the two drugs. Although clinically approved, the efficacy and safety profiles of pentamidine do not make this a favourable treatment choice in most contexts [2]; should AmB resistance emerge, however, hypersensitivity may make this an important option for control.

Most of the work we present here focuses on different routes to resistance via changes to sterol metabolism. Of note too, however, was the identification of a deletion to the gene encoding the miltefosine transporter in line AmBRB/cl2, with an accompanying drop in sensitivity to miltefosine. A previous study also identified mutations in the miltefosine transporter gene, a phospholipid translocase, associated with AmB resistance, thus further supporting a role for changes in the miltefosine transporter in altering sensitivity to AmB [20]. The authors of this study also found changes in membrane lipid composition in miltefosine transporter mutants, suggesting that this effect, or resultant influences on membrane fluidity, could affect the action of AmB at the membrane.

We observed and validated a role of genetic changes in two sterol biosynthesis enzymes, SMT and SC5D. Whilst the mechanism of AmB-ergosterol binding is not fully understood, *in vitro* analysis of the role of specific functional groups in ergosterol on AmB activity has revealed the importance both the C24-methyl group and 5(6)-7(8) double bond conjugation [16,17]. The former was suggested to promote Van der Waals interactions with AmB within the membrane [17], whereas the latter influences the rigidity of the ergosterol ring system, the structure thought to mediate Van der Waals interactions with the AmB heptaene moiety [46]. Therefore, in both cases, sterol changes could directly affect AmB binding, thus reducing its activity. Including CYP51, found in our previous work [21], we have now identified three genes in which mutations result in AmB resistance in *Leishmania*. It is unclear if this is an exhaustive list or if other genes will be involved, as found in fungi [47]. The identification of multiple routes to resistance is important because, although ergostane-type sterols are lost in each case, the point at which the pathway is mutated will lead to the accumulation of different sterols that might influence viability in different ways. The genes most frequently observed to mutate here encode SMT, and others have previously noted changes to the relative abundance of a pair of transcripts in *L*. *donovani* [15,22]. In wild-type promastigotes of both *L*. *donovani* and *L*. *mexicana*, expression is dominated by one type of transcript (*LmxM*.*36*.*2380* in *L*. *mexicana*), whilst in resistant lines, this transcript (or, more specifically, its 3’-UTR) is lost whilst the other (here, *LmxM*.*36*.*2390*) remains the same or is slightly increased. Here we demonstrate a role for genomic changes in these expression differences and propose models for these changes. In AmBRC/cl3 and AmBRD/cl2, loss of the *LmxM*.*36*.*2380* 3’-UTR is associated with a deletion event that bears the hallmarks of homologous recombination. During the preparation of this manuscript, loss of SMT expression was described in an AmB-resistant *L*. *infantum* line, accompanied by evidence of a deletion event that the authors suggested could arise due to homologous recombination [48]; the similarities with the model proposed here demonstrate that similar events can arise in multiple *Leishmania* species. In AmBRB/cl2, however, there is additional evidence of SIDER1-mediated amplification. The model proposed here, linear duplication followed by homologous recombination-mediated deletion, explains the data presented, including qPCR and WGS results, although it is possible that events happen as a single process, in which the process of SIDER1-mediated duplication promotes deletion events. Given the same elements appear to be involved in amplification in the previously selected amastigote line, it is feasible that these elements promote instability at this locus, thereby enabling other structural rearrangements. Whilst SIDER1 elements have previously been implicated in regulation of translation, but not transcript abundance [44,49], evidence of life cycle stage-dependent regulation of *LmxM*.*36*.*2380* but not *LmxM*.*36*.*2390* transcript abundance raises the possibility of a role for certain SIDER1 elements in regulating transcript stability. Moreover, a decrease in the ratio of *LmxM*.*36*.*2380* to *LmxM*.*36*.*2390* expression may necessitate that further loss of *LmxM*.*36*.*2390* expression is required for resistance in amastigotes. The F72C mutation in AmBRC/cl3 SMT subsequent to structural variation provides an example of such a change.

Fitness costs and the relative paucity of AmB resistance in fungi have led to the argument that AmB resistance may not pose a threat for treatment programmes. However, reports have indicated AmB treatment failure is appearing in *Leishmania* and at least one case of parasite resistance from a relapsing patient has been found [15]. Although selection of drug resistance in promastigotes poses a problem for the assessment of fitness costs, given that long-term growth *in vitro* often results in loss of infectivity, at least one of our selected lines, AmBRC/cl3, showed infectivity and growth were comparable to wild-type parasites, both in primary macrophages and *in vivo*. This differs from a recent observation that deletion of both SMT copies in *L*. *major* resulted in reduced virulence in a mouse model [50], which may suggest that retention of even small amounts of SMT activity is enough to maintain infectivity. AmB resistance was also maintained in intracellular amastigotes, which also underlines the importance of giving serious consideration to the risk of AmB resistance appearing in a clinical setting.

Due to structural instability of the genome environment in which *Leishmania* SMT genes reside, and a lack of evident fitness costs in selecting resistance through down-regulation of its expression, loss of SMT function should be of particular interest in efforts to monitor for emergent AmB resistance. Low-level resistance predisposes to higher-level resistance through accumulation of additional mutations. This happens in at least one line described here: higher levels of resistance are achieved in AmBRB through combination of SMT structural rearrangement following miltefosine transporter deletion. Disruption of the latter gene is already being selected in clinical populations due to widespread miltefosine administration, indicating that leishmanial parasites predisposed to selection for AmB resistance may already be in circulation.

## Supporting information

S1 Text Sequence of intergenic region between sterol C24-methyltransferase gene copies. Within the reference genome used here, there was evidence of incomplete assembly in the region between sterol C24-methyltransferase gene sequences *LmxM*.*36*.*2380* and *LmxM*.*36*.*2390*, notably a spacer region of 100 N residues. Therefore, we determined the sequence of the intergenic region by PCR amplification and Sanger sequencing. Below is the sequence derived. Note that positions 1–130 and 3019–3079 are derived from the 3’-end and 5’-end of flanking coding sequences.
(TXT)

S2 Text Sequences of SIDER1 elements promoting amplification at the SMT locus, and syntenic sequences in other Leishmania species. SIDER1 sequences. The first sequence (Intergenic SIDER1) is a 450 bp region derived from the SMT intergenic region sequence in S1 Text. The second is a region highly homologous to this from the intergenic region between *LmxM*.*36*.*2540* and *LmxM*.*36*.*2550*. The remaining sequences are regions of high sequence homology derived in syntenic positions from *L*. *major* and *L*. *infantum* (note that due to a fusion of chromosomes 36 and 20 in *L*. *mexicana*, these homologous sequences are located on chromosome 20 in *L*. *mexicana* and chromosome 36 in the other two species).
(TXT)

S1 Fig Growth curves of promastigotes. With a starting parasite density of 10^5^ cells/ml, three biological replicates were performed with counts for each replicate the average of two independent counts. Error bars represent standard deviation. The left panel depicts mean count values, the right panel log-transformed counts.
(PNG)

S2 Fig Ploidy in whole genome sequencing data. Ploidy ratios were calculated as the median length-normalised per-gene coverage for an individual chromosome in comparison to the median value across all chromosomes. These were then multiplied by two and rounded to give integral ploidy values, assuming basal diploidy.
(PNG)

S3 Fig Evidence of loss of heterozygosity associated with mutation. In each panel, heterozygous sites are plotted across a chromosome for one resistant line (top part) and wild-type (bottom part). Top: heterozygous sites in AmBRA/cl1 on chromosome 23, with the vertical red line marking the position of sterol C5-desaturase (*LmxM*.*23*.*1300*). Bottom: heterozygous sites in AmBRB/cl2 on chromosome 13, with the vertical red line marking the position of the miltefosine transporter (*LmxM*.*13*.*1530*). Note that in both cases, mutated or deleted genes fall within large regions where heterozygous sites are few or absent, indicating broad loss of heterozygosity events associated with homozygous mutation.
(PNG)

S4 Fig Alignment of sterol C5-desaturase sequences. Alignments were performed using Clustal Ω (https://www.ebi.ac.uk/Tools/msa/clustalo/). Sequences were derived from either TriTrypDB for kinetoplastid sequences (http://tritrypdb.org/tritrypdb/) or Uniprot for others. From top to bottom, species are as follows: *L*. *mexicana*, *L*. *major*, *L*. *donovani*, *L*. *braziliensis*, *Crithidia fasciculata*, *Trypanosoma*. *brucei*, *T*. *cruzi*, *S*. *cerevisiae*, *Homo sapiens* and *Arabidopsis thaliana*. The black arrow indicates the position of Gly139 (mutated to Arg in AmBRA/cl1), and black boxes represent the sites of conserved His residues, forming motifs that in the yeast enzyme have been implicated in catalysis or ligand binding.
(PNG)

S5 Fig Alignment of sterol C24-methyltransferase and related sequences. Alignments were performed using Clustal Ω. Sequences were derived from either TriTrypDB for kinetoplastid sequences or Uniprot for others. From top to bottom, species are: *L*. *mexicana*, *L*. *major*, *L*. *donovani*, *L*. *braziliensis*, *C*. *fasciculata*, *T*. *brucei*, *T*. *cruzi*, *S*. *cerevisiae* and *A*. *thaliana* (the *A*. *thaliana* enzyme is cycloartenol C24-methyltransferase). Black arrows indicate the position of variable sites F72, V131 and V321, the black box shows the putative sterol binding site as detected in yeast.
(PNG)

S6 Fig Chronological order of mutations arising during selection of resistance. During selection of resistance, parasites at different stages were subjected to cryopreservation. These were genotyped at the SMT locus by PCR amplification of the genes and Sanger sequencing. In the case of AmBRB, miltefosine transporter deletion was monitored by PCR amplification and gel electrophoresis. Graphs of AmB sensitivity show mean values, n = 4, with error bars representing standard deviation. Asterisks represent statistically significant (P < 0.05, two-tailed student’s *t*-test) differences from the previous subpassage. A) Selection of AmBRB. miltefosine transporter deletion occurs first, by 93 days (although it has already begun to disappear from the population by 75 days, reproducible across three biological replicates), followed by change in SMT genotype by 144 days. B) Selection of AmBRC. Genotyping of SMT shows that the G391A/G961A changes (associated with structural variation) occur by day 116 of selection, whereas the further homozygous mutation, T215G, occurs independently by day 213 of selection.
(PNG)

S7 Fig Distribution of expression changes in RNA-seq data. For each AmB-resistant line, the log_2_-transformed fold-change of each gene is plotted against mean counts. Significantly differentially expressed genes (corrected P value < 0.05) are plotted in red. This shows very large numbers of differentially expressed genes, but that these remain within ± 1 log_2_(FC) of wild-type. Generated using the R package DESeq2.
(PNG)

S8 Fig The effects of ploidy change on fold-change in gene expression. Violin plots show, for each AmB-resistant line, the distribution of fold-change in RNA expression in comparison to fold change in ploidy values as depicted in S2 Fig. Box plots are overlaid, showing the median and lower and upper quartiles. RNA fold-change values are calculated as the normalised fragment counts for individual clones divided by the mean normalised fragment counts for wild-type parasites (across three biological replicates). In all cases, the influence of ploidy was strongly significant (P < 10^−150^, Kruskal-Wallis test).
(PNG)

S9 Fig qRT-PCR in SMT ectopic overexpression lines. SMT expression was measured by qRT-PCR in lines ectopically expressing SMT. Plots show fold change compared to wild-type, error bars represent standard deviation, n = 3. Expression in all cases shows a significant increase in δCt vs wild-type (P < 0.05).
(PNG)

S10 Fig Images for gel electrophoresis experiments. All gels were 1% agarose in tris-acetate-EDTA buffer. In each case, flanking DNA standards are from the 1kb DNA ladder (Promega) (from top to bottom, in base pairs: 10,000; 8,000; 6,000; 5,000; 4,000; 3,000; 2,500; 2,000; 1,500; 1,000; 750; 500; 250). All PCR reactions used to generate amplicons are described in Methods. A) Amplification of *LmxM*.*36*.*2380* from genomic DNA using a forward primer at the start of the coding sequence and a reverse primer within the 3’-UTR specific to this gene copy. The amplicon associated with this genomic region is found only in wild-type and AmBRA/cl1 DNA. B) Amplification of *LmxM*.*36*.*2390* using a forward primer at the start of the coding sequence and a reverse primer within the 3’-UTR specific to this gene copy. This amplicon is detectable in all lines. C) Amplification of the intergenic region between SMT gene copies; primers bind within the SMT coding sequence, with the forward primer binding to the 3’-end and the reverse primer to the 5’-end. The amplicon associated with this genomic region is found only in wild-type and AmBRA/cl1 DNA. D) Amplification of the junction formed during SIDER1-mediated amplification; the forward primer binds within *LmxM*.*36*.*2540*, the reverse within the SMT coding sequence. This amplicon is found only in AmBRB/cl2.
(PNG)

S11 Fig Visualisation of genome sequencing coverage of the SMT locus. Visualisation was produced with the Integrative Genomics Viewer (http://software.broadinstitute.org/software/igv/) using whole genome sequencing data aligned to the *L*. *mexicana* reference genome with a corrected intergenic region. For each strain, the top part of the panel represents coverage, whereas the bottom part depicts individual reads. Grey blocks represent concordantly aligned reads with a mapping quality > 0, coloured blocks represent non-concordantly aligned reads. White-filled blocks represent reads with a mapping quality of 0. Many of these reads fall within the SMT coding sequences *LmxM*.*36*.*2380* and *LmxM*.*36*.*2390* (positions shown as blue blocks at the bottom of the plot), due to high homology of these sequences. Whilst data show continuous coverage in wild-type and AmBRA/cl1, there is a complete absence of coverage of the intergenic region in AmBRC/cl3 and AmBRD/cl2. AmBRB/cl2 has a small gap immediately downstream of *LxmM*.*36*.*2380*.
(PNG)

S12 Fig Visualisation of RNA-seq coverage of the SMT locus. Visualisation was produced with the Integrative Genomics Viewer (http://software.broadinstitute.org/software/igv/) using RNA-seq data aligned to the *L*. *mexicana* reference genome with a corrected intergenic region. See S11 Fig for full description. For wild-type and AmBRA/cl1, unique regions of coverage (grey blocks) can be seen immediately upstream (5’-UTRs) and downstream (3’-UTRs) for both SMT coding sequences. On the other hand, in AmBRC/cl3 and AmBRD/cl2, the 5’-UTR of *LmxM*.*36*.*2390* is absent, and there are no uniquely mapped reads in the 3’-UTR region of *LmxM*.*36*.*2380*. By contrast, AmBRB/cl2 has uniquely mapped reads in the 5’-UTR region of both coding sequences, but discontinuous coverage of the 3’-UTR of *LmxM*.*36*.*2380*.
(PNG)

S13 Fig Validation of the multigenic amplification in AmBRB/cl2. The duplication event suspected in AmBRB/cl2 based on WGS data was verified by qPCR to detect copy number changes in *LmxM*.*36*.*2400* and *LmxM*.*36*.*2540* (located at the start and the end of the amplified region), and *LmxM*.*36*.*2550* (located after the amplicon. The solid line indicates no change compared to wild-type genomic DNA, the dotted line a doubling of copy number in AmBRB/cl2 genomic DNA. P values for statistically significant changes are 8.81 x 10^−4^ and 0.00188 for *LmxM*.*36*.*2400* and *LmxM*.*36*.*2540*, respectively.
(PNG)

S1 Table AmB sensitivity in individual clones. For each resistant line, three individual clones were obtained by limiting dilution, and IC_50_ values (nM) were obtained for each (shown here ± standard deviation). Note that whilst all are statistically significantly higher than wild-type (P < 0.05, two-tailed student’s *t-*test), there are no significant differences between individual clones from the same line.
(XLSX)

S2 Table Identification of sterol-associated peaks in GC-MS data. Data are reported for sterols identified from *L*. *mexicana* sterol extracts, based on matches either to standards or to the NIST library of standards. The exception is ergosta-5,7,24(28)-trienol, which was identified based on previously reported literature.
(XLSX)

S3 Table Percentage sterol composition for first GC-MS experiment. The data here are used to generate Fig 1A. For individual replicates, initial percentage compositions were estimated, followed by omission of all peaks < 0.5% of total sterol content (giving these a 0 value) and recalculation of percentages. Mean values across three replicates are shown, ± standard deviation, n = 3.
(XLSX)

S4 Table Percentage sterol composition for second GC-MS experiment. The data here are used to generate Fig 4C. For individual replicates, initial percentage compositions were estimated, followed by omission of all peaks < 0.5% of total sterol content (giving these a 0 value) and recalculation of percentages. Mean values across three replicates are shown, ± standard deviation, n = 3.
(XLSX)

S5 Table Sensitivity to AmB and pentamidine in ectopic overexpression lines. IC_50_ values used in Fig 4A & 4B are shown, ± standard deviation. P_1_ values represent statistical significance of differences between individual lines and wild-type, P_2_ between ectopic overexpression lines and their parental AmB-resistant line (two-tailed student’s *t*-test). For AmB, n = 4, except for AmBRB/cl2 and AmBRD/cl2 (n = 6), and AmBRB/cl2 + miltefosine transporter, AmBRB/cl2 + SMT WT and AmBRD/cl2 + SMT WT (n = 8). For pentamidine, n = 5.
(XLSX)

S6 Table Primers used for qPCR experiments. (XLSX)

S7 Table Primers used for amplification of genomic regions. (XLSX)

S8 Table Additional primers used for Sanger sequencing. (XLSX)

S1 Data Differential expression analysis of RNA-seq data from wild-type and AmB-resistant promastigotes. (XLSX)
