# Supplementary figures and images for: Genomic instability at the locus of sterol C24-methyltransferase promotes amphotericin B resistance in Leishmania parasites
Source: PLoS Negl Trop Dis. 2019 Feb 4;13(2):e0007052. doi: 10.1371/journal.pntd.0007052 (PMC6375703; doi:10.1371/journal.pntd.0007052)

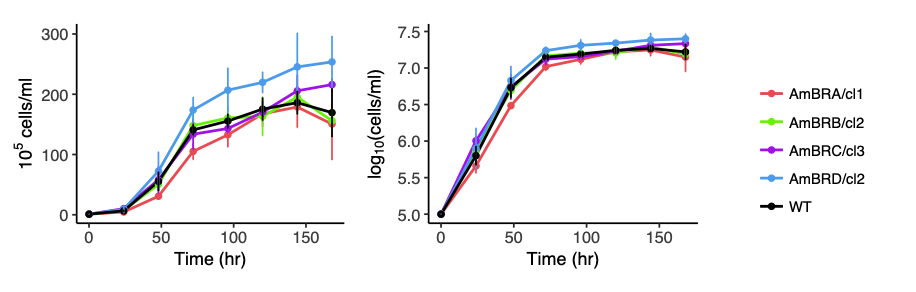

Supplement: S1 Fig — With a starting parasite density of 105 cells/ml, three biological replicates were performed with counts for each replicate the average of two independent counts. Error bars represent standard deviation. The left panel depicts mean count values, the right panel log-transformed counts. (PNG) [file pntd.0007052.s003.png]

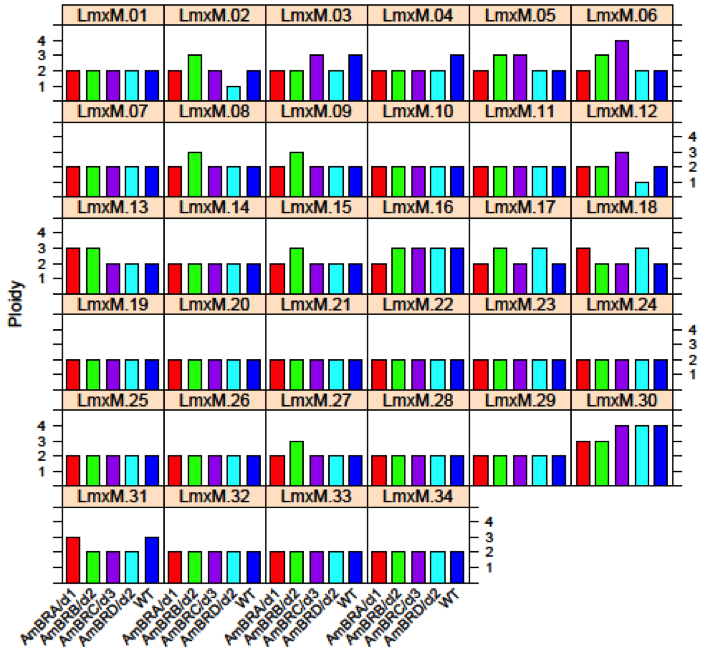

Supplement: S2 Fig — Ploidy ratios were calculated as the median length-normalised per-gene coverage for an individual chromosome in comparison to the median value across all chromosomes. These were then multiplied by two and rounded to give integral ploidy values, assuming basal diploidy. (PNG) [file pntd.0007052.s004.png]

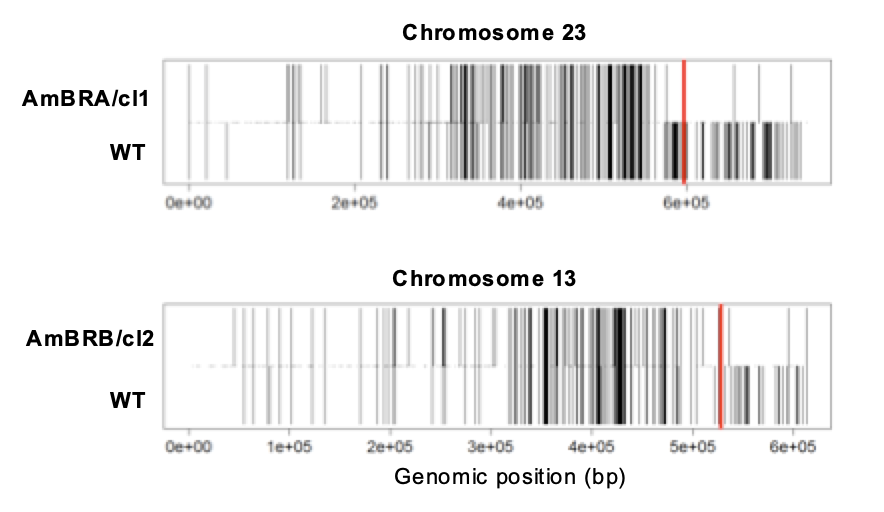

Supplement: S3 Fig — In each panel, heterozygous sites are plotted across a chromosome for one resistant line (top part) and wild-type (bottom part). Top: heterozygous sites in AmBRA/cl1 on chromosome 23, with the vertical red line marking the position of sterol C5-desaturase (LmxM.23.1300). Bottom: heterozygous sites in AmBRB/cl2 on chromosome 13, with the vertical red line marking the position of the miltefosine transporter (LmxM.13.1530). Note that in both cases, mutated or deleted genes fall within large regions where heterozygous sites are few or absent, indicating broad loss of heterozygosity events associated with homozygous mutation. (PNG) [file pntd.0007052.s005.png]

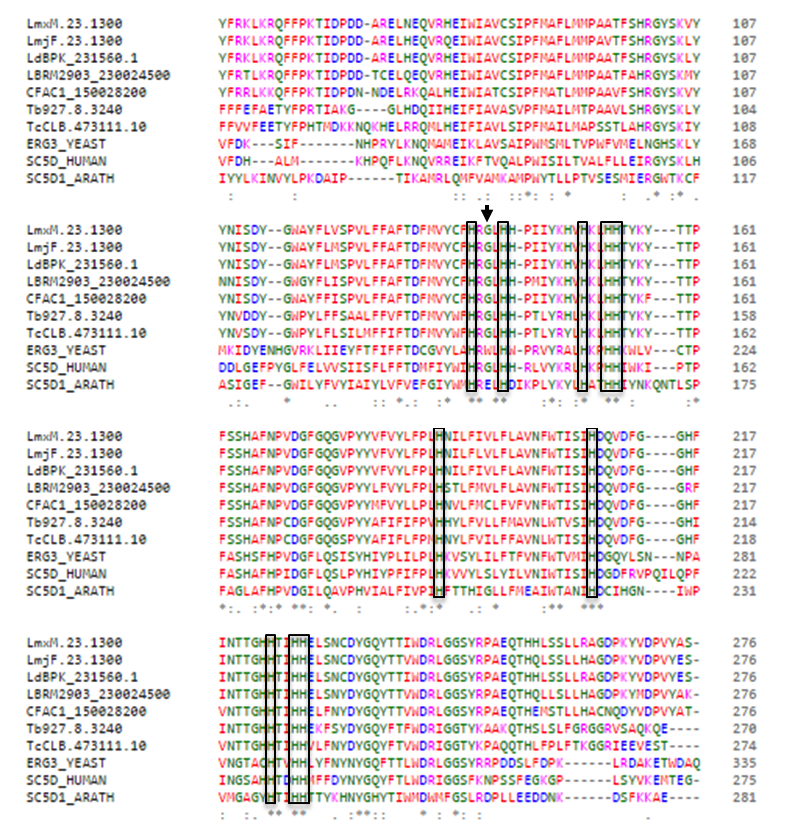

Supplement: S4 Fig — Alignments were performed using Clustal Ω (https://www.ebi.ac.uk/Tools/msa/clustalo/). Sequences were derived from either TriTrypDB for kinetoplastid sequences (http://tritrypdb.org/tritrypdb/) or Uniprot for others. From top to bottom, species are as follows: L. mexicana, L. major, L. donovani, L. braziliensis, Crithidia fasciculata, Trypanosoma. brucei, T. cruzi, S. cerevisiae, Homo sapiens and Arabidopsis thaliana. The black arrow indicates the position of Gly139 (mutated to Arg in AmBRA/cl1), and black boxes represent the sites of conserved His residues, forming motifs that in the yeast enzyme have been implicated in catalysis or ligand binding. (PNG) [file pntd.0007052.s006.png]

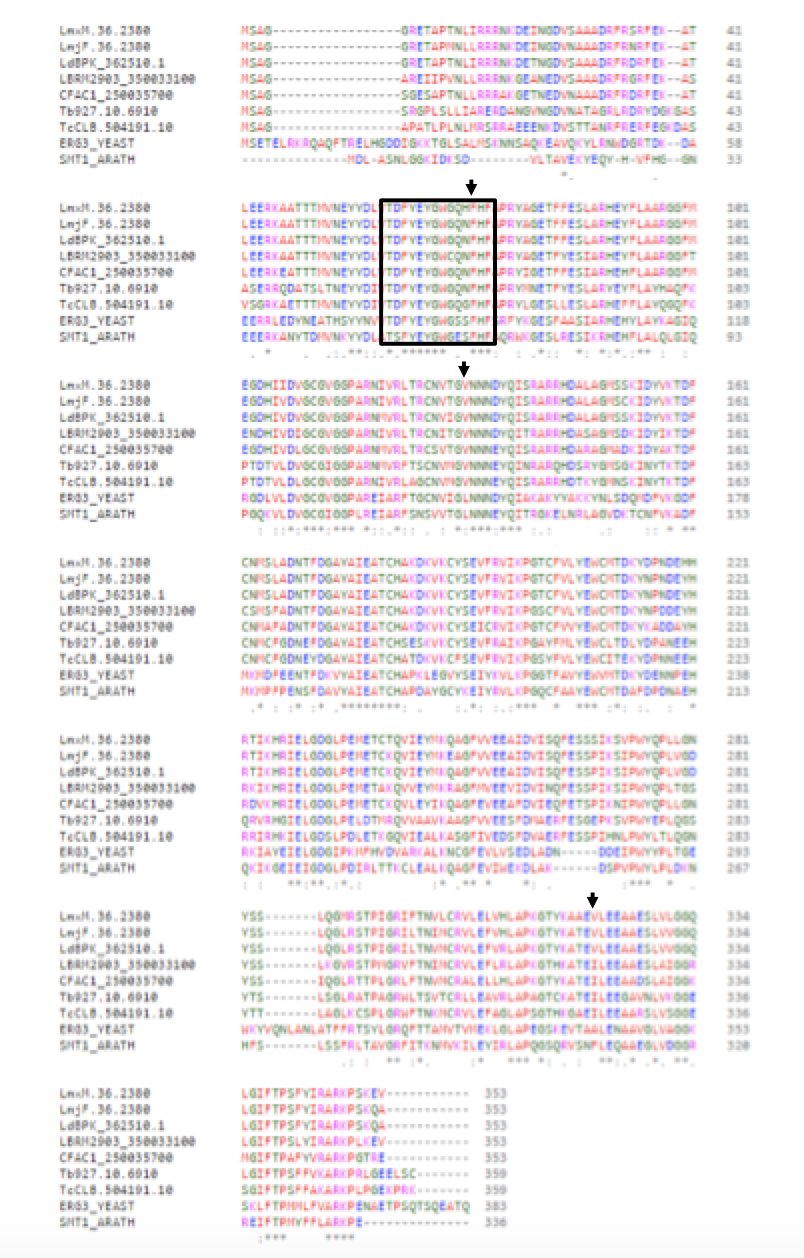

Supplement: S5 Fig — Alignments were performed using Clustal Ω. Sequences were derived from either TriTrypDB for kinetoplastid sequences or Uniprot for others. From top to bottom, species are: L. mexicana, L. major, L. donovani, L. braziliensis, C. fasciculata, T. brucei, T. cruzi, S. cerevisiae and A. thaliana (the A. thaliana enzyme is cycloartenol C24-methyltransferase). Black arrows indicate the position of variable sites F72, V131 and V321, the black box shows the putative sterol binding site as detected in yeast. (PNG) [file pntd.0007052.s007.png]

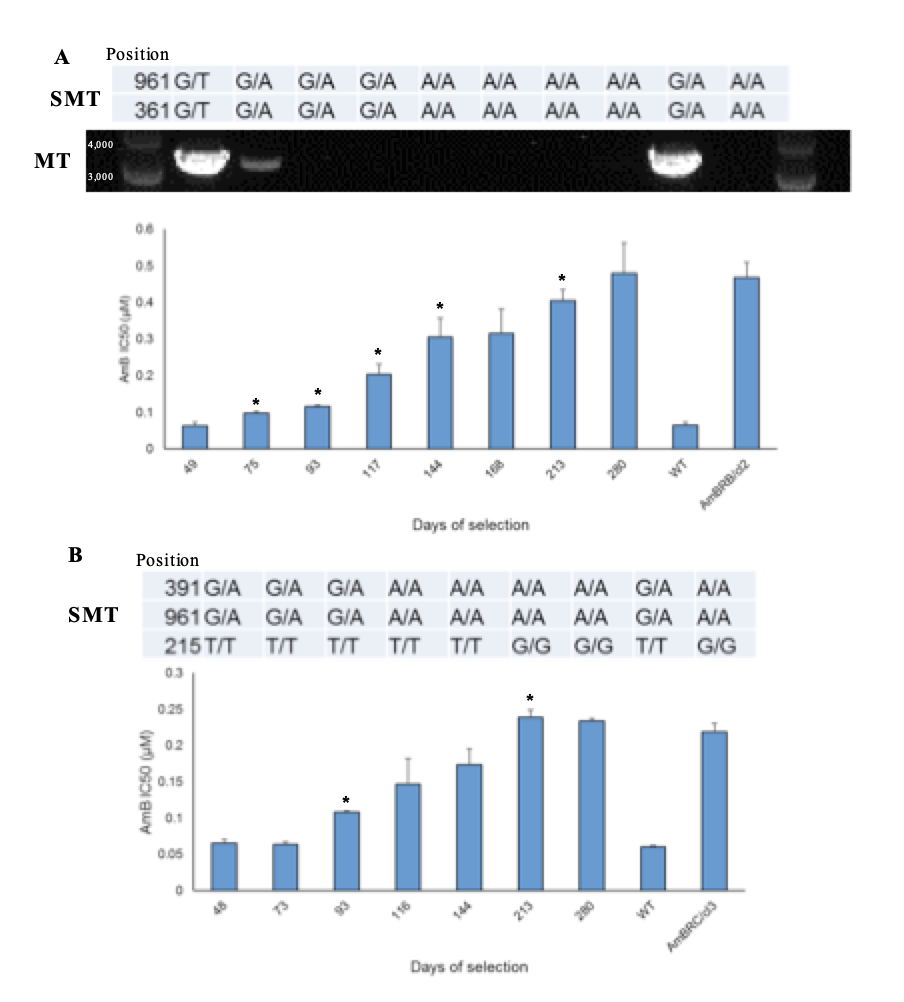

Supplement: S6 Fig — During selection of resistance, parasites at different stages were subjected to cryopreservation. These were genotyped at the SMT locus by PCR amplification of the genes and Sanger sequencing. In the case of AmBRB, miltefosine transporter deletion was monitored by PCR amplification and gel electrophoresis. Graphs of AmB sensitivity show mean values, n = 4, with error bars representing standard deviation. Asterisks represent statistically significant (P < 0.05, two-tailed student’s t-test) differences from the previous subpassage. A) Selection of AmBRB. miltefosine transporter deletion occurs first, by 93 days (although it has already begun to disappear from the population by 75 days, reproducible across three biological replicates), followed by change in SMT genotype by 144 days. B) Selection of AmBRC. Genotyping of SMT shows that the G391A/G961A changes (associated with structural variation) occur by day 116 of selection, whereas the further homozygous mutation, T215G, occurs independently by day 213 of selection. (PNG) [file pntd.0007052.s008.png]

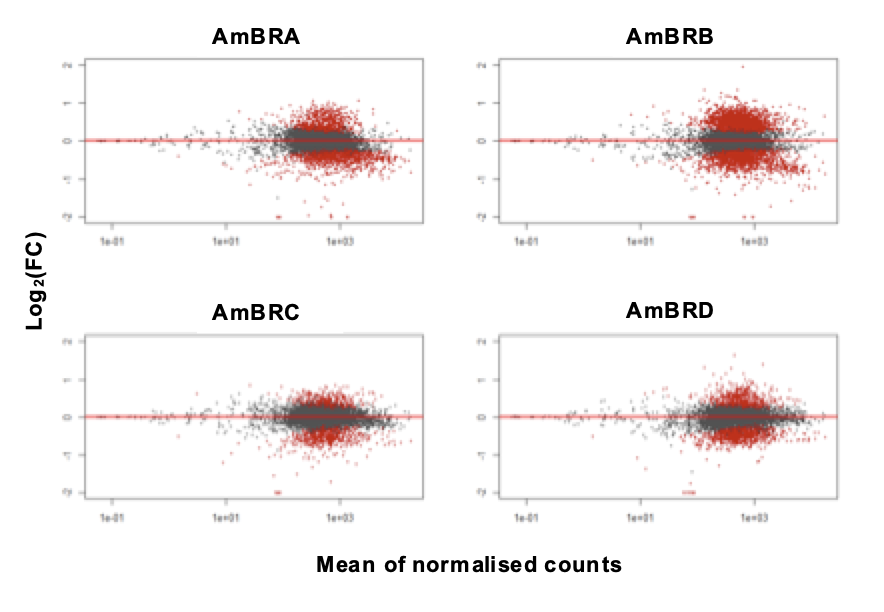

Supplement: S7 Fig — For each AmB-resistant line, the log2-transformed fold-change of each gene is plotted against mean counts. Significantly differentially expressed genes (corrected P value < 0.05) are plotted in red. This shows very large numbers of differentially expressed genes, but that these remain within ± 1 log2(FC) of wild-type. Generated using the R package DESeq2. (PNG) [file pntd.0007052.s009.png]

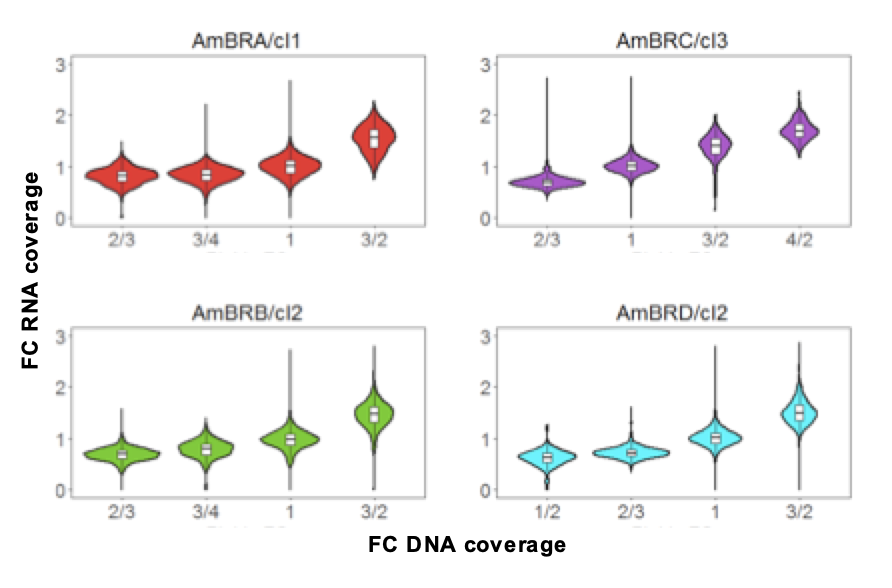

Supplement: S8 Fig — Violin plots show, for each AmB-resistant line, the distribution of fold-change in RNA expression in comparison to fold change in ploidy values as depicted in S2 Fig. Box plots are overlaid, showing the median and lower and upper quartiles. RNA fold-change values are calculated as the normalised fragment counts for individual clones divided by the mean normalised fragment counts for wild-type parasites (across three biological replicates). In all cases, the influence of ploidy was strongly significant (P < 10−150, Kruskal-Wallis test). (PNG) [file pntd.0007052.s010.png]

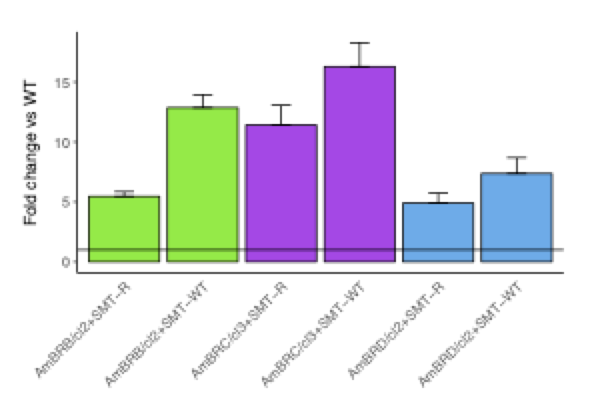

Supplement: S9 Fig — SMT expression was measured by qRT-PCR in lines ectopically expressing SMT. Plots show fold change compared to wild-type, error bars represent standard deviation, n = 3. Expression in all cases shows a significant increase in δCt vs wild-type (P < 0.05). (PNG) [file pntd.0007052.s011.png]

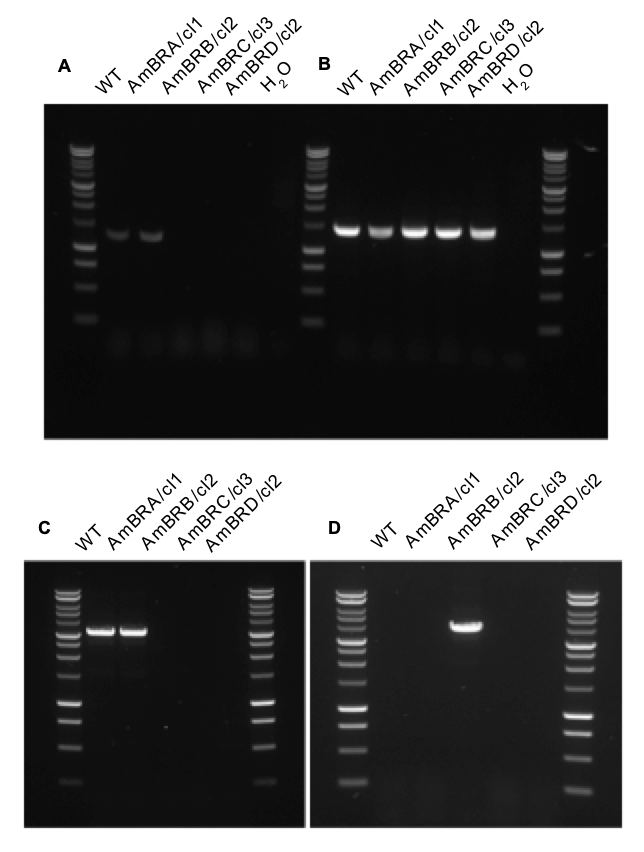

Supplement: S10 Fig — All gels were 1% agarose in tris-acetate-EDTA buffer. In each case, flanking DNA standards are from the 1kb DNA ladder (Promega) (from top to bottom, in base pairs: 10,000; 8,000; 6,000; 5,000; 4,000; 3,000; 2,500; 2,000; 1,500; 1,000; 750; 500; 250). All PCR reactions used to generate amplicons are described in Methods. A) Amplification of LmxM.36.2380 from genomic DNA using a forward primer at the start of the coding sequence and a reverse primer within the 3’-UTR specific to this gene copy. The amplicon associated with this genomic region is found only in wild-type and AmBRA/cl1 DNA. B) Amplification of LmxM.36.2390 using a forward primer at the start of the coding sequence and a reverse primer within the 3’-UTR specific to this gene copy. This amplicon is detectable in all lines. C) Amplification of the intergenic region between SMT gene copies; primers bind within the SMT coding sequence, with the forward primer binding to the 3’-end and the reverse primer to the 5’-end. The amplicon associated with this genomic region is found only in wild-type and AmBRA/cl1 DNA. D) Amplification of the junction formed during SIDER1-mediated amplification; the forward primer binds within LmxM.36.2540, the reverse within the SMT coding sequence. This amplicon is found only in AmBRB/cl2. (PNG) [file pntd.0007052.s012.png]

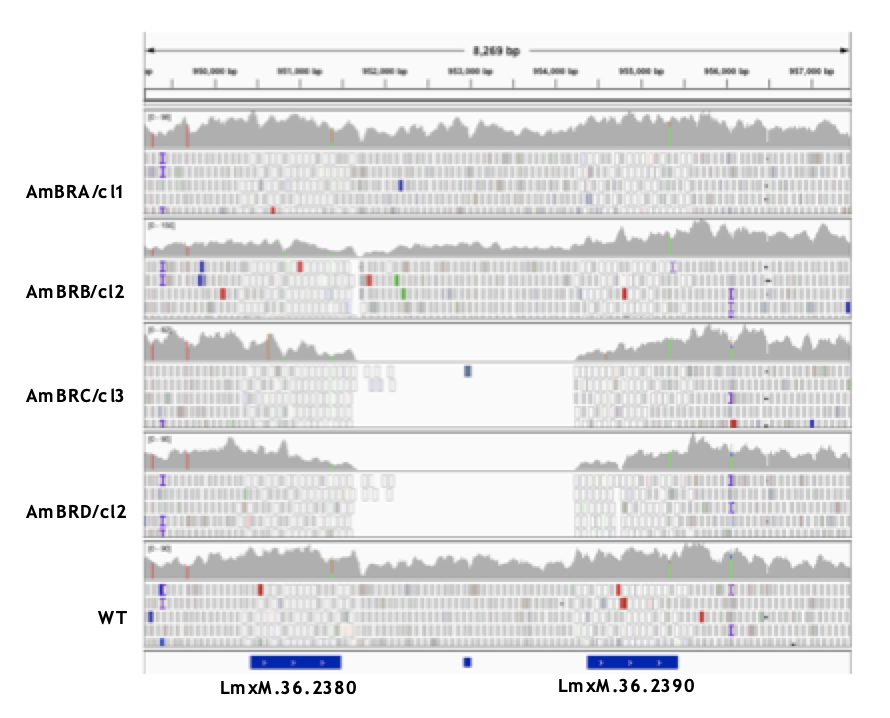

Supplement: S11 Fig — Visualisation was produced with the Integrative Genomics Viewer (http://software.broadinstitute.org/software/igv/) using whole genome sequencing data aligned to the L. mexicana reference genome with a corrected intergenic region. For each strain, the top part of the panel represents coverage, whereas the bottom part depicts individual reads. Grey blocks represent concordantly aligned reads with a mapping quality > 0, coloured blocks represent non-concordantly aligned reads. White-filled blocks represent reads with a mapping quality of 0. Many of these reads fall within the SMT coding sequences LmxM.36.2380 and LmxM.36.2390 (positions shown as blue blocks at the bottom of the plot), due to high homology of these sequences. Whilst data show continuous coverage in wild-type and AmBRA/cl1, there is a complete absence of coverage of the intergenic region in AmBRC/cl3 and AmBRD/cl2. AmBRB/cl2 has a small gap immediately downstream of LxmM.36.2380. (PNG) [file pntd.0007052.s013.png]

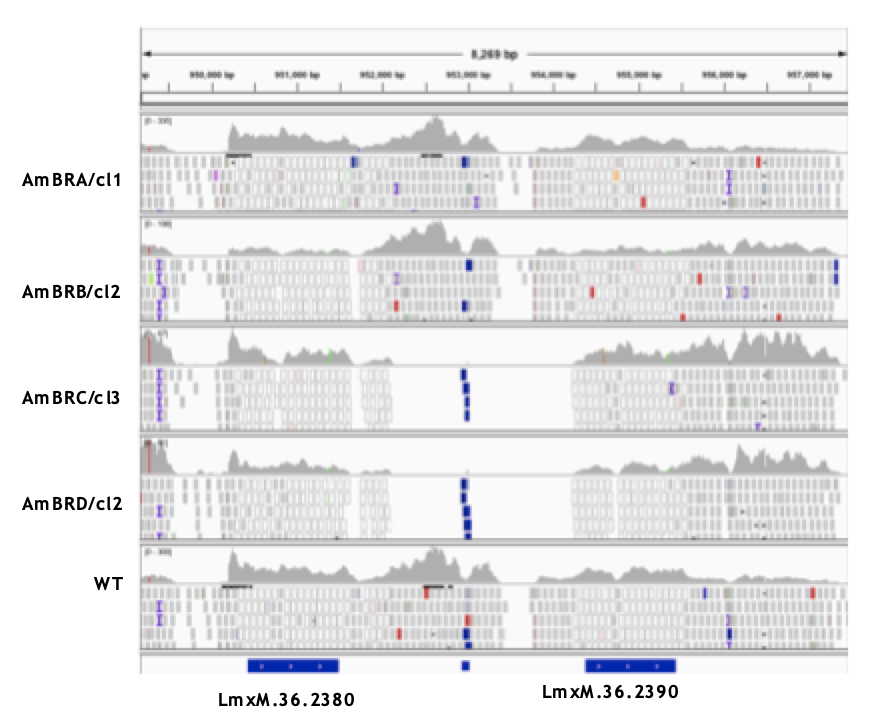

Supplement: S12 Fig — Visualisation was produced with the Integrative Genomics Viewer (http://software.broadinstitute.org/software/igv/) using RNA-seq data aligned to the L. mexicana reference genome with a corrected intergenic region. See S11 Fig for full description. For wild-type and AmBRA/cl1, unique regions of coverage (grey blocks) can be seen immediately upstream (5’-UTRs) and downstream (3’-UTRs) for both SMT coding sequences. On the other hand, in AmBRC/cl3 and AmBRD/cl2, the 5’-UTR of LmxM.36.2390 is absent, and there are no uniquely mapped reads in the 3’-UTR region of LmxM.36.2380. By contrast, AmBRB/cl2 has uniquely mapped reads in the 5’-UTR region of both coding sequences, but discontinuous coverage of the 3’-UTR of LmxM.36.2380. (PNG) [file pntd.0007052.s014.png]

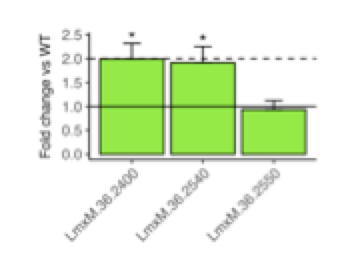

Supplement: S13 Fig — The duplication event suspected in AmBRB/cl2 based on WGS data was verified by qPCR to detect copy number changes in LmxM.36.2400 and LmxM.36.2540 (located at the start and the end of the amplified region), and LmxM.36.2550 (located after the amplicon. The solid line indicates no change compared to wild-type genomic DNA, the dotted line a doubling of copy number in AmBRB/cl2 genomic DNA. P values for statistically significant changes are 8.81 x 10^−4^ and 0.00188 for LmxM.36.2400 and LmxM.36.2540, respectively. (PNG) [file pntd.0007052.s015.png]
